# Fourier power, subjective distance, and object categories all provide plausible models of BOLD responses in scene-selective visual areas

**DOI:** 10.3389/fncom.2015.00135

**Published:** 2015-11-05

**Authors:** Mark D. Lescroart, Dustin E. Stansbury, Jack L. Gallant

**Affiliations:** ^1^Helen Wills Neuroscience Institute, University of California, BerkeleyBerkeley, CA, USA; ^2^Vision Science Program, University of California, BerkeleyBerkeley, CA, USA; ^3^Department of Psychology, University of California, BerkeleyBerkeley, CA, USA

**Keywords:** scene perception, fMRI, voxel-wise modeling, encoding models, neuroscience, vision

## Abstract

Perception of natural visual scenes activates several functional areas in the human brain, including the Parahippocampal Place Area (PPA), Retrosplenial Complex (RSC), and the Occipital Place Area (OPA). It is currently unclear what specific scene-related features are represented in these areas. Previous studies have suggested that PPA, RSC, and/or OPA might represent at least three qualitatively different classes of features: (1) 2D features related to Fourier power; (2) 3D spatial features such as the distance to objects in a scene; or (3) abstract features such as the categories of objects in a scene. To determine which of these hypotheses best describes the visual representation in scene-selective areas, we applied voxel-wise modeling (VM) to BOLD fMRI responses elicited by a set of 1386 images of natural scenes. VM provides an efficient method for testing competing hypotheses by comparing predictions of brain activity based on encoding models that instantiate each hypothesis. Here we evaluated three different encoding models that instantiate each of the three hypotheses listed above. We used linear regression to fit each encoding model to the fMRI data recorded from each voxel, and we evaluated each fit model by estimating the amount of variance it predicted in a withheld portion of the data set. We found that voxel-wise models based on Fourier power or the subjective distance to objects in each scene predicted much of the variance predicted by a model based on object categories. Furthermore, the response variance explained by these three models is largely shared, and the individual models explain little unique variance in responses. Based on an evaluation of previous studies and the data we present here, we conclude that there is currently no good basis to favor any one of the three alternative hypotheses about visual representation in scene-selective areas. We offer suggestions for further studies that may help resolve this issue.

## Introduction

fMRI experiments have shown that natural scene perception activates several distinct functional areas in the human cerebral cortex. These include the Parahippocampal Place Area (PPA), Retrosplenial Complex (RSC), and the Occipital Place Area (OPA, also known as the Temporal Occipital Sulcus or TOS) (Aguirre et al., 1998; Epstein and Kanwisher, 1998; Maguire, 2001; Nasr et al., 2011; Dilks et al., 2013). Which specific scene-related features are represented in these areas has been the subject of substantial debate.

Several qualitatively different scene-related features have been proposed to be represented in scene-selective areas. Some studies have suggested that these areas represent simple 2D features related to the Fourier power spectrum (Rajimehr et al., 2011; Nasr and Tootell, 2012; Nasr et al., 2014; Watson et al., 2014). Others have argued that PPA, RSC, and OPA represent features related to 3D spatial structure, such as expanse or openness (Kravitz et al., 2011; Park et al., 2011), the distance from objects in a scene to an observer (Amit et al., 2012; Park et al., 2015), or the size of objects in a scene (Cate et al., 2011; Konkle and Oliva, 2012). A third position is that scene-selective areas represent information about the semantic categories of natural scenes or their constituent objects (Walther et al., 2009, 2011; Huth et al., 2012; Stansbury et al., 2013).

Previous studies have not resolved which of these hypotheses provides the best account of the representation of natural scenes in scene-selective areas. One reason that this has been a difficult issue to resolve is that almost every previous study of scene-selective cortical areas has used stimuli that were pre-selected or manipulated to maximize variation in specific stimulus features of interest. Consequently, different experiments use different stimuli, and thereby sample different ranges of variation in stimulus features. If the brain operated according to purely linear mechanisms, this would not cause any problems for scientific interpretation of the results. However, feature tuning in the human visual system is conferred by nonlinear mechanisms that operate at all levels of the visual hierarchy (Van Essen et al., 1992). In such a nonlinear system, responses to a limited range of stimulus variation cannot necessarily be used to infer responses to stimulus variation outside that range (Wu et al., 2006; Gallant et al., 2012). Thus, any experiment that constrains stimulus variation may fail to characterize nonlinear tuning properties for stimuli (or stimulus features) that fall outside the experiment's pre-selected stimulus set.

The most straightforward way to probe the visual system in an ecologically valid range is to use a broad distribution of natural images as stimuli. The human visual system is exquisitely tuned to the statistical variance and covariance of features in natural images (Field, 1987; Simoncelli and Olshausen, 2001). Thus, one efficient way to determine what features are represented in scene-selective areas is to record brain activity elicited by a wide range of natural scenes, extract features from the stimulus images that reflect the various hypotheses, and then determine which features best account for the measured brain activity (Naselaris et al., 2009, 2012; Nishimoto et al., 2011; Stansbury et al., 2013).

In this study, we analyzed BOLD fMRI responses to a large set of natural photographs to determine which features of natural scenes are represented in PPA, RSC, and OPA. We employed a voxel-wise modeling (VM) approach in which we directly compared predictive models based on three different classes of scene-related features: 2D features derived from the Fourier power spectrum of each scene, the distance to salient objects in each scene, and semantic categories of the constituent objects in each scene. For each class of features, we defined a feature space to formalize each alternative hypothesis in quantitative terms.

To estimate the relationship between each feature space and measured BOLD responses, we used linear regression to fit each feature space to the fMRI data recorded from each voxel in the posterior part of the brain (encompassing the visual cortex). Each feature space and its associated β weights constitute an encoding model that maps a stimulus onto brain responses. We evaluated each model based on how accurately it predicted BOLD responses in a separate validation data set. Finally, we applied a variance partitioning analysis to determine whether different models predict unique or shared variance in BOLD responses.

## Methods

The data used for this experiment came from previously published studies from our laboratory. The four subjects in this experiment are the same four subjects as in Stansbury et al. (2013). Data for two of these subjects (subjects 1 and 2) were originally collected for Naselaris et al. (2012). Here we provide a brief description of the stimuli, subjects, data collection, and image response estimation. For full details, see Stansbury et al. (2013).

### fMRI data acquisition and preprocessing

All fMRI data were collected at the UC Berkeley Brain Imaging Center using a 3 Tesla Siemens Tim Trio MR Scanner (Siemens, Germany). Data were collected from each of four human subjects (1 female) while they viewed 1386 natural images. The data were collected over six or seven scanning sessions for each subject, and the total scan time per subject was 4 h and 53 min. Voxels were approximately 2.25 × 2.25 × 2.99 mm, and the repetition time (TR) was approximately 2 s. The fMRI scan protocol used for subject one was slightly different from the protocol used for the others; see Stansbury et al. (2013) for full details. Anatomical scans were acquired for each subject using a T1-weighted magnetization-prepared rapid gradient echo (MP-RAGE) sequence. All subjects gave their written informed consent to participate, and the experimental protocol was approved by the UC Berkeley Committee for the Protection of Human Subjects.

Freesurfer was used to automatically extract cortical surfaces from the T1-weighted scans (Dale et al., 1999). These surfaces were manually edited to improve the match to the anatomical data. Surface flattening and visualization were performed with Freesurfer and custom python code (Gao et al., 2015; available at http://github.com/gallantlab/pycortex).

Functional MRI data were preprocessed using custom Matlab (R2014a, MathWorks) code and SPM8 (http://www.fil.ion.ucl.ac.uk/spm/software/spm8/). For each subject, data were motion corrected and coregistered to the first volume collected. The motion correction and coregistration transformations were concatenated, and the data were re-sliced only once. Data were divided into two separate subsets: one used for model estimation and one used for model validation. The preprocessed BOLD responses were de-convolved into a unique hemodynamic response per voxel and a unique response amplitude per image per voxel. Response amplitudes for the validation data were estimated slightly differently from the method in Stansbury et al. (2013) in order to obtain an estimate of the noise in each voxel (see Noise Ceiling Estimation section below).

### Stimuli

The experimental stimuli consisted of 1386 photographs of natural scenes. Most of the photographs used in this study were selected first from a collection of 4000 labeled images curated by the Lotus Hill Institute (Wuhan, China). The labels provided by the Lotus Hill Institute were used to re-label all images as containing (or not containing) each of four non-mutually exclusive superordinate categories: *animal, human, manmade*, and *natural*. Animals and humans were prioritized because animacy was a principal feature of interest in Naselaris et al. (2012). An additional 242 images were downloaded from Google Images, in order to increase the number of scenes containing both animals and humans. Finally, 1386 images were randomly chosen from the full set of 4242 images, such that approximately the same number of images had the labels *animal* and *human*, and (independently) such that approximately the same number of images had the labels *natural* and *manmade*. Thus, images were not specifically selected based on the features of interest in this study. All four subjects saw the same 1386 stimulus images. Figure S02 shows all 126 validation images shown to all four subjects.

Images subtended 20° × 20° of visual angle (500 × 500 pixels). Each image presentation consisted of five brief flashes in 1 s, followed by 3 s of isoluminant gray screen. The 1260 images in the estimation data set were repeated twice each. The 126 images in the validation data set were repeated 12 times each. During the experiment subjects maintained steady fixation on a small (0.2° × 0.2°) square that changed colors at 3 Hz. Subjects were instructed to try to understand each scene as it was presented, but had no explicit task besides maintaining fixation. Stimulus presentation and all statistical analyses were conducted using custom Matlab (R2014a, MathWorks) and python code.

### Feature spaces used for voxel-wise encoding models

In voxel-wise modeling, a *feature space* is a quantification of the features of a stimulus that are hypothesized to be related to brain responses (Naselaris et al., 2011; Gallant et al., 2012). For this study we created three different feature spaces: a Fourier power feature space, subjective distance feature space, and an object category feature space. Each feature space embodies a different hypothesis about which features are represented in scene-selective areas.

#### Fourier power feature space

**Figure 1:**
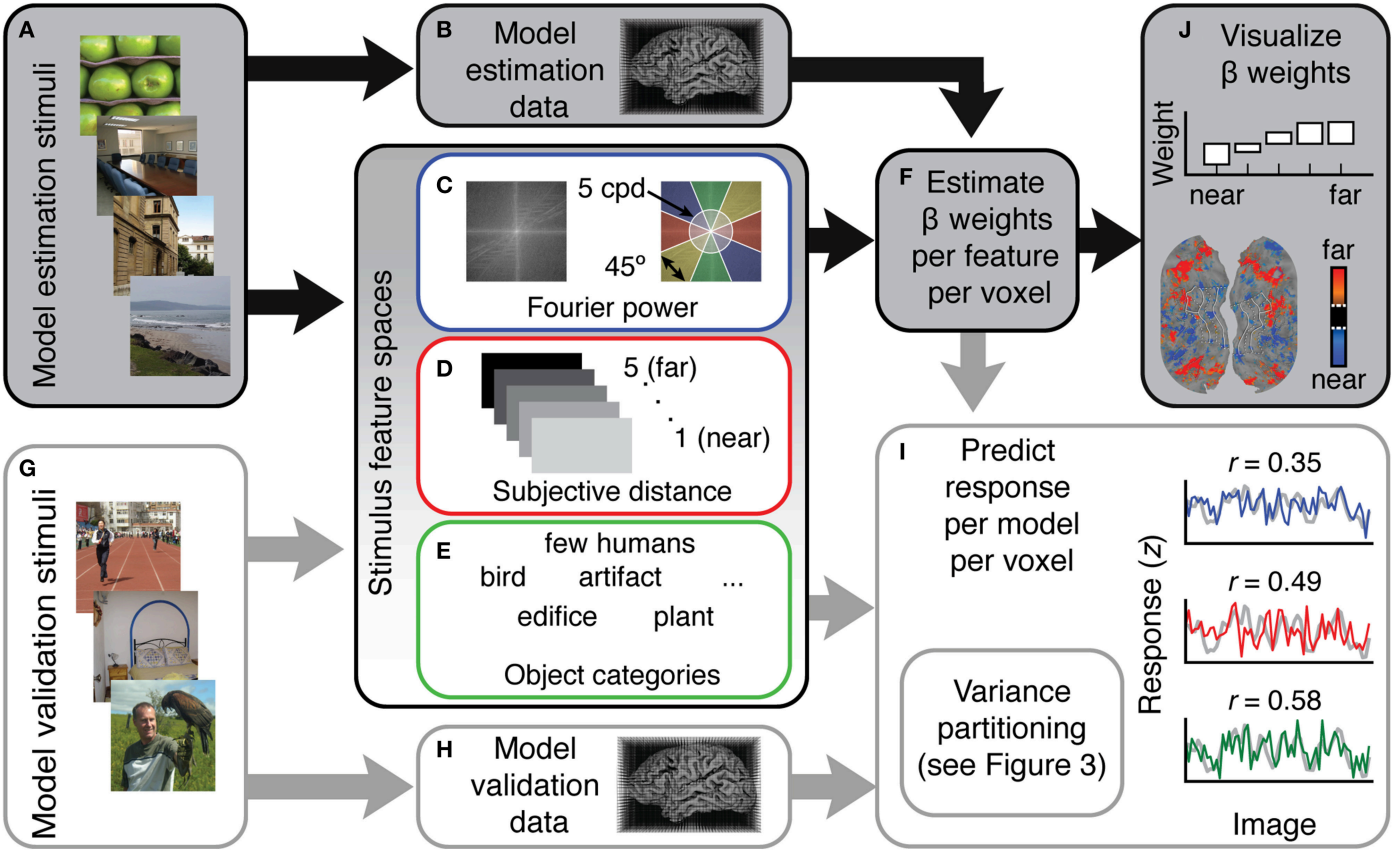
**Overview of the voxel-wise modeling (VM) procedure used in this study**. **(A)** Human subjects were shown 1260 natural images while **(B)** fMRI data were recorded. **(C–E)** These data were modeled as a function of three different feature spaces. Each feature space reflects a different hypothesis about which features are represented in scene-selective areas. **(C)** For the Fourier power mode, the feature space was computed by taking the Fourier transform of each stimulus image and then averaging the amplitude spectrum over the orientation and spatial frequency bins shown at right. **(D)** For the subjective distance model, the feature space consisted of ratings from three humans who judged whether the main content of each stimulus scene was (1) < 2 ft away, (2) < 4 ft away, (3) < 20 ft away, (4) < 100 ft away, and (5) >100 ft away. **(E)** For the semantic category model the feature space consisted of labels from three human raters who labeled the objects in each stimulus image using 19 semantic labels. **(F)** Ordinary least squares regression was used to find a set of weights (β) that map the features in each model onto the BOLD responses in each voxel. Each feature space and its associated β weights constitute a different encoding model. **(G)** In order to validate the models in an independent data set, the same subjects were shown a different set of 126 images while **(H)** fMRI responses were collected. **(I)** To assess model accuracy, the β weights estimated from the training data were used to predict responses in this withheld model validation data set. **(J)** To reveal patterns of tuning in the features quantified by each different model, pre-specified *t* contrasts were computed between β weights in each model and projected onto the cortical surface, and β weights were averaged over voxels in different regions of interest and plotted.

To parameterize variation in spatial frequency energy at different orientations, we created a Fourier power feature space. First, the color images were converted to Commission Internationale de l'Éclairage L^*^A^*^B^*^ color space, and the luminance layer was extracted. A 2D Fourier transform was computed for each luminance image. The amplitude spectrum for each image was divided into eight bins: one high-frequency and one low-frequency bin at each of four orientations (0, 45, 90, and 135°). The divide between high and low frequency bins was set at five cycles/degree, as in Rajimehr et al. (2011) and Nasr and Tootell (2012). A schematic of the Fourier domain bins is shown in Figure 1C. Fourier power was averaged over each bin for each image. To reduce correlations between Fourier power bins, each bin in each image was divided by the L2 norm of all bins for that image. The L2 norm itself was retained as a separate feature reflecting the overall spatial frequency energy in each image. Thus, the final Fourier power feature space consisted of nine feature channels: one total spatial frequency energy channel (i.e., the L2 norm), four low spatial frequency channels, and four high spatial frequency channels (One can think of each feature channel as a separate column in a regression design matrix). To match the range of variation in the Fourier power feature channels to the range of the *z*-scored BOLD responses, each feature channel was *z*-scored separately across all images.

#### Subjective distance feature space

To parameterize distance in each scene, we created a subjective distance feature space based on human distance judgments. Human raters were instructed to estimate the distance to the main content (the most salient or subjectively important objects) in each of the 1386 stimulus images. The determination of the main content of each image was left to the discretion of each rater, so these distance ratings were inherently subjective. For each image, raters chose one of five roughly logarithmically spaced distance bins: (1) extreme closeup, ~1–2 ft., (2) arm's length, ~3–4 ft., (3) nearby/same room, < 20 ft., (4) semi-distant, < 100 ft., or (5) far away, >100 ft. Raters viewed each image for 300 ms before making each rating. These brief durations approximated the brief image presentation time used in the fMRI experiment. Raters had the option to repeat an image if they felt they had not adequately understood it, but repeated viewing was discouraged. Three different raters provided distance judgments. Two of them were also subjects in the fMRI experiment. The ratings produced by the three raters were consistent: the correlations between the three raters' distance ratings were 0.845, 0.857, and 0.861. The median distance rating for each image across all three raters was used to code the features. The final subjective distance feature space consisted of five mutually exclusive binary feature channels (one for each distance bin).

#### Object category feature space

To parameterize semantic variation in our stimulus images, we used an object category feature space based on human-assigned labels indicating the presence of objects or other scene elements (such as land, water, and sky) in each image. This feature space was originally created for an earlier study (Naselaris et al., 2012). For a full description of the labeling process, see the original paper. Briefly, 15 human raters assigned natural language labels to each object in each image. These labels were binned into 19 categories: creepy animal (e.g., insects, snakes, and reptiles), bird, fish, water mammal, land mammal, many humans, few humans, vehicle, artifact, text, prepared food, fruit vegetable, other plants, furniture, sky, water, land, part of building, and edifice. These categories span several superordinate categories known to be represented in higher-order visual areas (animate/inanimate, large/small, human/non-human). Thus, the full object category feature space consisted of 19 non-exclusive binary feature channels, each indicating the presence of a different object category in each stimulus image. In previous work models based on this feature space have been shown to provide accurate predictions of BOLD responses in several higher-order visual areas (Naselaris et al., 2012). This object category model also provides a simple approximation of the WordNet (Miller, 1995) feature space used to model BOLD data in Huth et al. (2012).

These three feature spaces were chosen as simple examples of three broader classes of hypotheses regarding the representation in scene-selective areas: that scene-selective areas represent low-level, image-based features, 3D spatial information, and categorical information about objects and scenes. Many other implementations of these broad hypotheses are possible, but an exhaustive comparison of all of the potential models is impractical at this time. Instead, here we focus on just three specific feature spaces that each capture qualitatively different information about visual scenes and that are simple to implement. We emphasize simplicity here for instructional purposes, for ease of interpretation, and to simplify the model fitting procedures and variance partitioning analysis presented below.

### Model fitting and evaluation

We used ordinary least squares regression to find a set of weights (β) that map the feature channels onto the estimated BOLD responses for the model estimation data (Figure 1H). Separate β weights were estimated for each feature channel and for each voxel. Each β weight reflects the strength of the relationship between variance in a given feature channel and variance in the BOLD data. Thus, each β weight also reflects the response that a particular feature is likely to elicit in a particular voxel. The model β weights as a whole demonstrate the *tuning* of a voxel or an area to specific features within the feature space for that model. The full set of β weights for all feature channels for a voxel constitute an encoding model for that voxel. Note that many previous fMRI studies from our laboratory (Nishimoto et al., 2011; Huth et al., 2012; Stansbury et al., 2013) have used ridge regression or another regularized regression procedure to produce voxel-wise encoding models that have the highest possible prediction accuracy. We did not use regularized regression in the current study because the use of regularization complicates interpretation of the variance partitioning analysis described below. Furthermore, the number of features in each model fit here was small relative to the amount of data collected, so regularization did not improve model performance.

Many studies describe the tuning of voxels across the visual cortex by computing *t* contrasts between estimated regression β weights for each voxel (Friston et al., 1994). To facilitate comparison of our results to the results of several such studies, we computed three *t* contrasts between β weights in each of our three models. Each contrast was computed for all cortical voxels. Using the β weights in the Fourier power model, we computed a contrast of cardinal vs. oblique high-frequency orientations (Nasr and Tootell, 2012). This contrast was specifically (+ high freq 0° + high freq 90° – high freq 45° – high freq 135°) (see **Figure 4** for feature naming scheme). Using the β weights in the subjective distance model, we computed a contrast of far vs. near distances (+ v. far + distant – near – closeup) (Amit et al., 2012; Park et al., 2015). Using the β weights in the object category model, we computed a contrast of people vs. buildings (+ few people –0.5 edifice –0.5 part of building) (Epstein and Kanwisher, 1998). Since these contrasts were computed for every voxel in the brain, the *p*-values for each *t* contrast were adjusted using False Discovery Rate (FDR) with an α level of 0.05 to correct for multiple comparisons (Benjamini and Yekutieli, 2001).

To evaluate the accuracy of each model, we used the model fit to each voxel to predict BOLD responses of the same voxel in the validation data set. Prediction accuracy was assessed by computing Pearson's product-moment correlation (*r*) between the predicted response and the validation response estimated for each voxel. To convert prediction accuracy to an estimate of the variance explained, we squared the prediction accuracy (*r*) for each model in each voxel value while maintaining its sign (David and Gallant, 2005).

### Noise ceiling estimation

Noise in the validation data set will nearly always bias prediction accuracy downward, and the magnitude of this bias may differ across voxels. This makes raw prediction accuracy difficult to interpret: for any given voxel, imperfect predictions may be caused by a flawed model, measurement noise, or both. To correct this downward bias and to exclude noisy voxels from further analyses, we used the method of Hsu et al. (Hsu et al., 2004; Huth et al., 2012) to estimate a noise ceiling (γ) for each voxel in our data. The noise ceiling is the amount of response variance in the validation data that could theoretically be predicted by the perfect model.

Noise ceiling estimation requires repeated measurement of responses to the same stimulus (Hsu et al., 2004). Thus, we estimated 11 different responses to each of our validation stimuli for each voxel. We split the validation data into 11 partially overlapping blocks. Each block contained two presentations of each stimulus image. The first block contained the first and second presentations of each image, the second block contained the second and third presentations of each image, and so on. For each block, the BOLD data were de-convolved into a unique hemodynamic response per voxel and a unique response amplitude per image per voxel. This procedure resulted in 11 different estimates of the response to each of our validation images for each voxel. These 11 validation image response estimates were used to compute the noise ceiling (γ) for each voxel.

**Figure 2:**
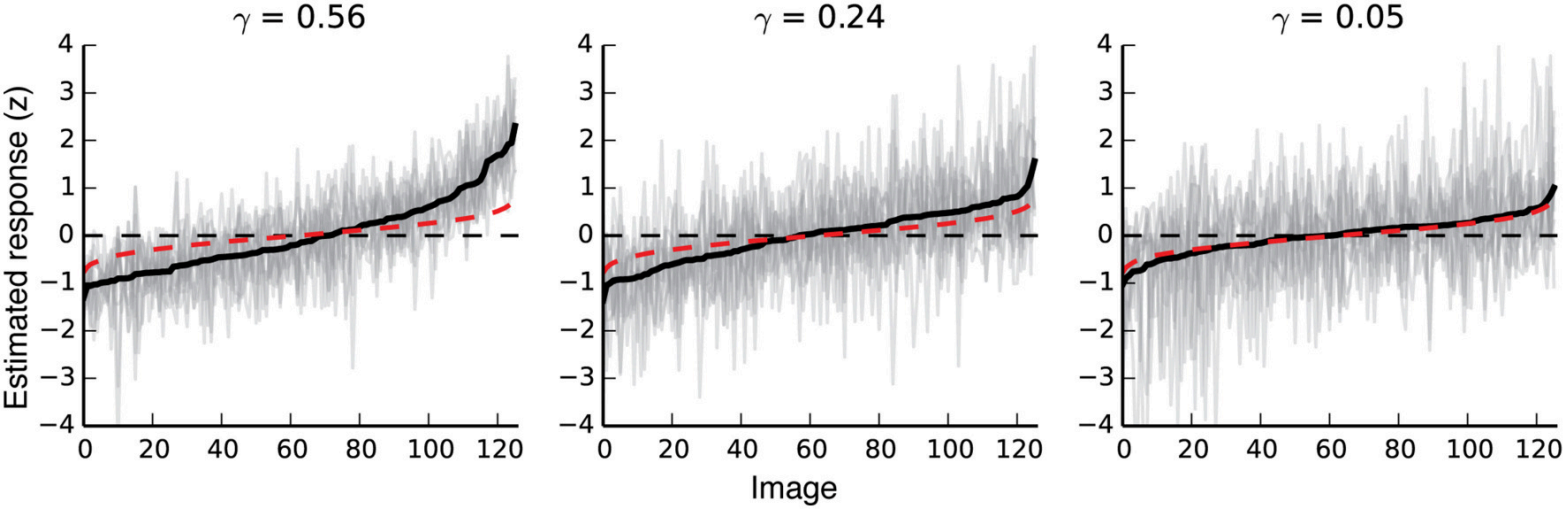
**Response variability in voxels with different noise ceilings**. The three plots show responses to all validation images for three different voxels with noise ceilings that are relatively high, moderate, and just above chance. The far-right plot shows the response variability for a voxel that meets our minimum criterion for inclusion in further analyses. Black lines show the mean response to each validation image. For each plot, images are sorted left to right by the average estimated response for that voxel. The 11 gray lines in each plot show 11 separate estimates of response amplitude per image for each voxel. Red dotted lines show random responses (averages of 11 random Gaussian vectors sorted by the mean of the 11 random vectors). Note that even random responses will deviate slightly from zero at the high and low ends, due to the bias induced by sorting the responses by their mean.

γ can be interpreted as a measure of signal repeatability. If the same stimuli reliably elicit similar responses, γ is high (near one); if not, it is low (near zero). To give a sense for this metric, Figure 2 shows estimated responses for three voxels with noise ceilings (γ-values) that are relatively high, average, and just above chance. Estimated γ-values were used to select voxels for all analyses presented in this paper. Voxels with noise ceilings greater than γ = 0.04 [a value corresponding to bootstrapped *p*(γ) < 0.01 for a single voxel] were retained, and all others were discarded before further analysis. In auditory cortex, where the signal should not be strongly related to the stimuli in this experiment, this threshold retains approximately five percent of the voxels. Figure S01 shows the absolute number of voxels kept, the percent of voxels kept, and the mean γ-value for each region of interest for each subject.

The noise ceiling was also used to normalize prediction accuracy in order to estimate the proportion of potentially explainable response variance that is actually explained by the models. The square root of the noise ceiling (γ^1/2^) gives the theoretical maximum correlation between predicted and observed responses for each voxel. Following Hsu et al. (2004), all estimates of prediction accuracy were divided by γ^1/2^. Estimates of variance explained were divided by γ. Note that very low noise ceilings can result in divergent normalized correlation estimates. For example, for γ = 0.0001 and *r* = 0.07, the normalized value of *r* would be 0.07/0.0001^1/2^ = 7. Our voxel selection criterion allows us to avoid such divergent estimates, since all voxels with low γ-values are discarded.

### Model comparison

To determine which features are most likely to be represented in each visual area, we compared the predictions of competing models on a separate validation data set reserved for this purpose. First, all voxels whose noise ceiling failed to reach significance [γ > 0.04, *p*(γ) > 0.01 uncorrected] were discarded. Next, the predictions of each model for each voxel were normalized by the estimated noise ceiling for that voxel. The resulting values were converted to *z* scores by the Fisher transformation (Fisher, 1915). Finally, the scores for each model were averaged separately across each ROI.

For each ROI, a permutation analysis was used to determine the significance of model prediction accuracy (vs. chance), as well as the significance of *differences* between prediction accuracies for different models. For each feature space, the feature channels were shuffled across images. Then the entire analysis pipeline was repeated (including fitting β weights, predicting validation responses, normalizing voxel prediction correlations by the noise ceiling, Fisher *z* transforming normalized correlation estimates, averaging over ROIs, and computing the average difference in accuracy between each pair of models). This shuffling and re-analysis procedure was repeated 10,000 times. This yielded a distribution of 10,000 estimates of prediction accuracy for each model and for each ROI, under the null hypothesis that there is no systematic relationship between model predictions and fMRI responses. Statistical significance was defined as any prediction that exceeded 95% of all of the permuted predictions (*p* = 0.05), calculated separately for each model and ROI. Note that different numbers of voxels were included in each ROI, so different ROIs had slightly different significance cutoff values. Significance levels for differences in prediction accuracy between models were determined by taking the 95th percentile of the distribution of differences in prediction accuracy between randomly permuted models (*p* = 0.05).

### Variance partitioning

**Figure 3:**
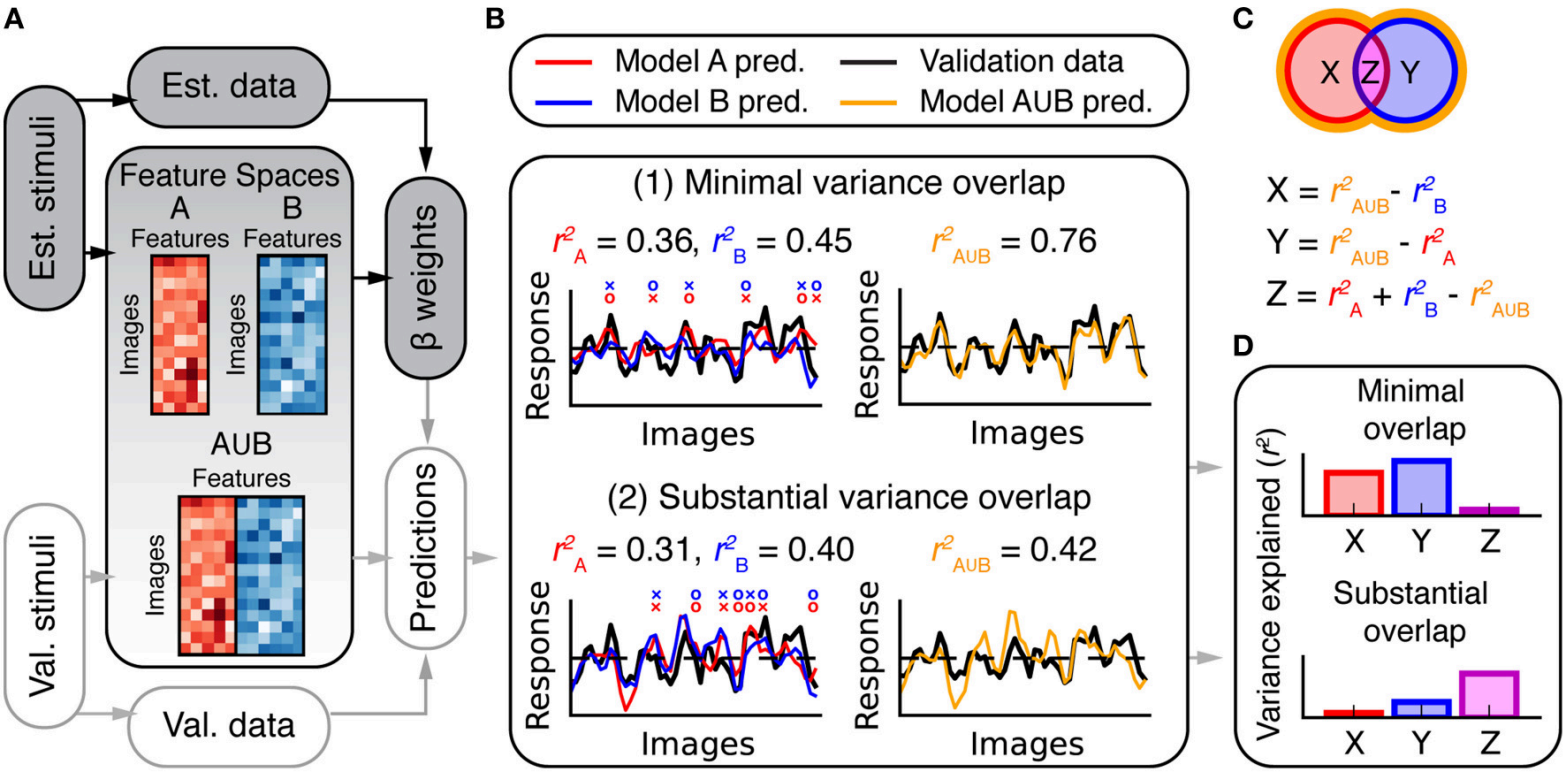
**Overview of variance partitioning analysis**. Variance partitioning determines what fraction of variance in BOLD responses is shared between two models. **(A)** To estimate the amount of shared variance between each pair or trio of feature spaces, all pairs or trios of feature spaces were concatenated (in the features dimension) and the resulting combined feature spaces were fit to the data and used to compute predictions of the validation data. **(B)** Two simulated models that predict (1) independent variance and (2) shared variance. In (1), each model tends to make accurate predictions (o marks) where the other fails (× marks). Consequently, the combined model (A∪B) performs well. In (2), both models succeed and fail for the same images (that is, the predictions are correlated). Consequently, the combined model does not perform better than the individual models. The total variance explained by models A and B can be subdivided into the partitions shown in the Venn diagram in **(C)**. Each partition corresponds to variance explained by: (X) only model A, (Y) only model B, and (Z) both A and B (shared variance). The variance explained by the combined model ($r_{\text{A}\cup \text{B}}^{2}$) provides an estimate of the convex hull of the Venn diagram (shown by the orange border). Thus, X, Y, and Z can be computed as shown. **(D)** Bar graphs of the values for X, Y, and Z computed for the two cases in **(B)**.

Estimates of prediction accuracy can determine which of several models best describes BOLD response variance in a voxel or area. However, further analysis is required to determine whether two models each explain unique or shared variance in BOLD responses. For example, consider two hypothetical models A and B. Suppose that model A makes slightly more accurate predictions than does model B for a given voxel. One possibility is that the variance explained by model B is a subset of the larger variance explained by model A. Another possibility is that model B explains a unique and complementary component of the response variance that is not explained by model A (For example, even if model B is worse overall it might make more accurate predictions than model A for a subset of images). Figure 3B shows two simulated examples in which competing models explain unique and shared response variance.

We performed a variance partitioning analysis (Figure 3) to determine the extent to which the three models in this study predict unique or shared components of the response variance in each scene-selective area. First, β weights were fit to each feature space independently (Figure 1). Then, feature spaces were concatenated in the features dimension (Figure 3A) for each possible pair or trio of feature spaces (Fourier power ∪ subjective distance, Fourier power ∪ semantic categories, subjective distance ∪ semantic categories, and Fourier power ∪ subjective distance ∪ semantic categories). For example, the feature space matrix resulting from the concatenation of all three models had 33 feature channels (nine from the Fourier power model, five from the subjective distance model, and 19 from the semantic category model). Each concatenated feature space was fit to the data for each voxel, and used to predict responses in the validation data for each voxel. Prediction accuracy was converted to variance explained by squaring the prediction correlation while maintaining its sign.

For pairwise variance partitioning, the unique and shared variance explained by each model or pair of models was computed according to the equations in Figure 3C. Similarly straightforward arithmetic was used to perform three-way variance partitioning to compute each element of the Venn diagram in **Figure 9**. For example, the unique variance explained by the semantic category model was estimated as the difference between variance explained by the full, 3-part concatenated model (Fourier power ∪ subjective distance ∪ semantic category) and the 2-part concatenation of the Fourier power and subjective distance models (Fourier power ∪ subjective distance).

### Evaluation of correlations between stimulus features

One risk associated with the use of natural images as stimuli is that features in different feature spaces may be correlated. If some of the features in different feature spaces are correlated, then models based on those feature spaces are more likely to generate correlated predictions. And if model predictions are correlated, the variance explained by the models will be shared (see Figure 3). To explore the consequences of correlated features, we computed the Pearson correlation (*r*) between all features in the Fourier power, subjective distance, and object category feature spaces. To determine whether the correlations between features that we measure in our stimulus set are general to many stimulus sets, we also explored feature correlations in two other stimulus sets (from Kravitz et al., 2011 and Park et al., 2015—see Supplementary Methods).

Non-zero correlations between a subset of the features in different feature spaces may or may not give rise to models that share variance. Two partially correlated feature spaces are most likely to lead to models that share variance if the feature channels that are correlated are also correlated with brain activity.

For example, imagine two simple feature spaces A and B, each consisting of three feature channels. A and B are used to model some brain activity, Y. Suppose that the first feature channel in A (A_1_) is correlated with the first feature channel in B (B_1_) at *r* = 0.5, and that the other feature channels (A_2_, A_3_, B_2_, and B_3_) are not correlated with each other or with Y at all. If A_1_ and B_1_ are both correlated with Y, then a linear regression that fits A and B to Y will assign relatively high β weights to A_1_ and B_1_ in the fit models (call the fit models M_A_ and M_B_). This, in turn, will make the predictions of M_A_ and M_B_ more likely to be correlated. Thus, M_A_ and M_B_ will be more likely to share variance.

Now, imagine a second case. Suppose instead that A_1_ and B_1_ are correlated with one another but neither A_1_ nor B_1_ is correlated with Y. Suppose that the other feature channels in A and B are correlated with Y to varying degrees. In this case, A_1_ and B_1_ will be assigned small β weights when A and B are fit to Y. The small β weights on A_1_ and B_1_ will mean that those two channels (the correlated channels) will not substantially affect the predictions of M_A_ and M_B_. Thus, in this case, the predictions of M_A_ and M_B_ will not be correlated, and M_A_ and M_B_ will each explain unique variance. These two simple thought experiments illustrate how the emergence of shared variance depends on correlations between feature channels and the β weights on those feature channels.

To illustrate how the correlations between features in this specific study interact with the voxel-wise β weights for each feature to produce shared variance across models, we conducted a simulation analysis. In brief, we simulated voxel responses based on the real feature values and two sets of β weights and performed variance partitioning on the resulting data. First, we used the concatenated stimulus feature spaces (X) and a set of semi-random weights (β) to generate simulated voxel data, according to the regression equation:

(1) $${\text{Y}}_{\text{sim}}=\text{X}\beta +\varepsilon$$

ε is Gaussian noise ~N(0,1). To assure that the simulated data had approximately the same signal-to-noise ratio as the fMRI data in our experiment, we modified the basic regression equation to scale the noise according to a distribution of expected correlations (ρ), thus:

(2) $${\text{Y}}_{\text{sim}}=\rho \text{X}\beta +{\left(1-\rho^{2}\right)}^{1/2}\varepsilon$$

We simulated the same number of voxels that we measured in all the scene-selective areas in all four subjects (761 voxels). We used the following procedure to assure that the simulation β weights were plausible given the covariance structure of the different feature spaces. First, we generated 761 different sequences of Gaussian random noise. Then we used ordinary least squares regression to fit β weights for each feature channel to the noise sequences. This resulted in 761 sets of β weights that map the feature spaces onto random data. Since ordinary least squares regression uses the feature covariance matrix to estimate β weights, the β weights generated by this procedure are guaranteed to be plausible given the covariance of the feature channels. Each set of semi-random β weights was then used to generate a simulated voxel timecourse according to Equation (2) above. We also created a second set of simulated data, based on the actual β weights we estimated for each of the 761 voxels in the experiment.

To illustrate how the specific β weights (the real β weights or the semi-random β weights) affected estimates of shared variance, we applied the same variance partitioning analysis that we applied to the fMRI data to both sets of simulated data. Note that the results of the variance partitioning of the simulated data based on the real β weights should match the results of the variance partitioning of the BOLD data. We include these results to show that our simulation procedure is operating as expected, and to demonstrate that any difference between the two simulations is a result of differences in the weights, and not anything to do with the simulation procedure.

### Functional area localizers

Visual areas in retinotopic visual cortex as well as functionally defined category-selective visual areas were identified in separate scan sessions using conventional methods (Spiridon et al., 2006; Hansen et al., 2007). Scene-selective areas PPA, RSC, and OPA were all defined by a contrast of places vs. objects. The Fusiform Face Area (FFA) was defined by a contrast of faces vs. objects. The boundaries of each area were hand drawn on the cortical surface at the locations at which the *t* statistic for the contrast of places vs. objects changed most rapidly.

## Results

To investigate how natural scenes are represented in scene-selective areas in the human brain, we analyzed BOLD fMRI signals evoked by a large set of natural images (These data were collected for two studies from our laboratory that were published previously: Naselaris et al., 2012 and Stansbury et al., 2013). We tested three specific hypotheses about scene representation in these areas that have been proposed in previous studies: that scene selective areas represent Fourier power, subjective distance, and object categories. To formalize each of these hypotheses, we defined three feature spaces that quantified three classes of features: Fourier power at different frequencies and orientations, distance to the salient objects in each scene, and the semantic categories of objects and other components of each scene. To determine the relationship between each feature space and brain activity, we used ordinary least squares regression to estimate sets of β weights that map each feature space onto the BOLD fMRI responses in the model estimation data set.

We present our results in four sections. First, we examine the tuning revealed by the estimated model β weights in V1, the FFA, the PPA, RSC, and the OPA. Second, we estimate the importance of each feature space by predicting responses in a withheld data set. Third, we evaluate whether each of these feature spaces predicts unique or shared response variance in the fMRI data. Finally, we investigate the correlations between features in the Fourier power, subjective distance, and object category feature spaces.

### Voxel-wise model β weights replicate tuning patterns described in previous studies

**Figure 4:**
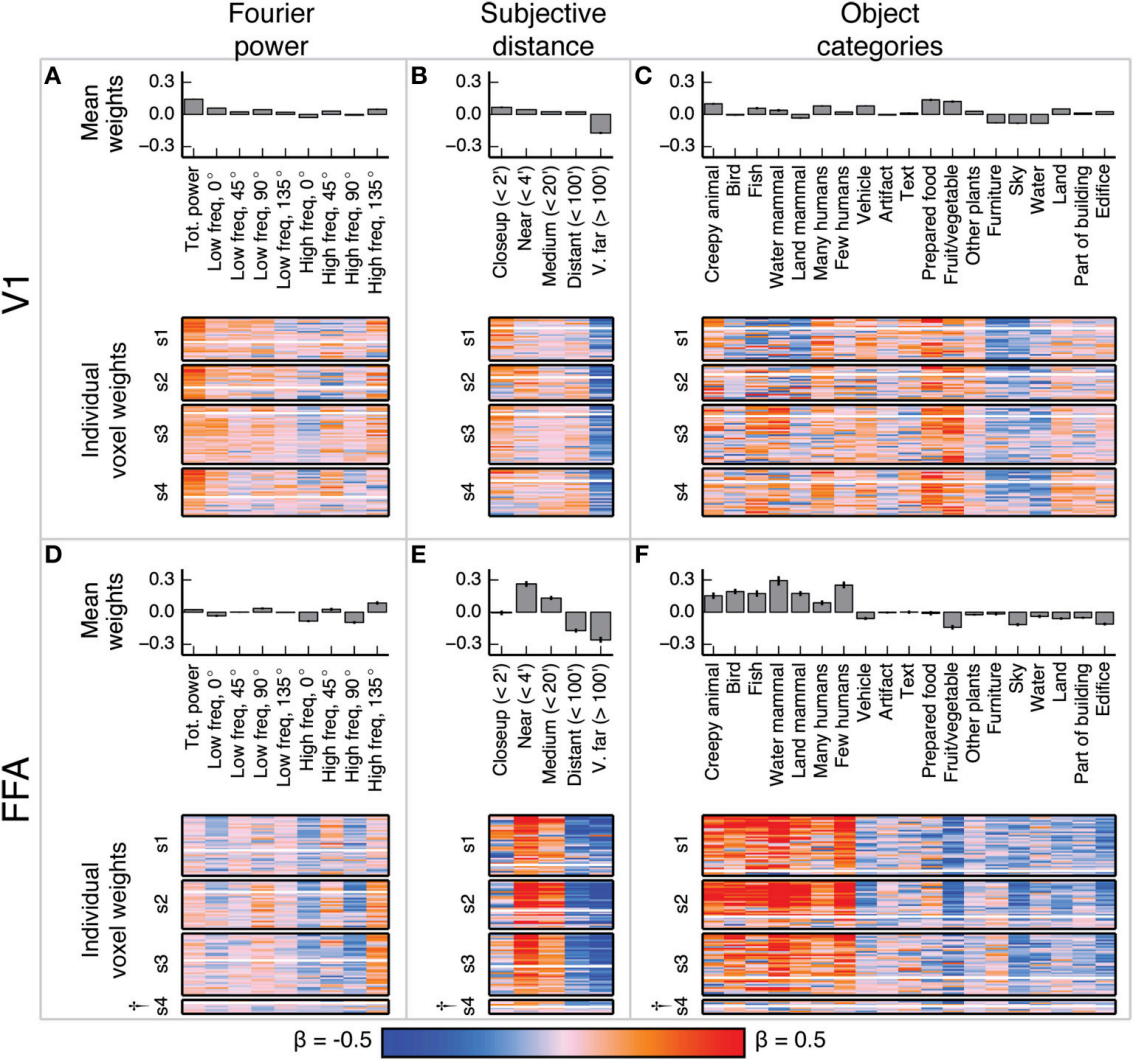
**Voxel-wise model β weights for all models for all voxels in V1 and FFA**. **(A)** Model β weights for the Fourier power model for V1. The image in the lower part of the panel shows the weight for every voxel in V1 that met our selection criterion [γ > 0.04, *p*(γ) < 0.01, see Methods]. Voxels are separated by subject (s1–s4), and the relative size of each subject's section indicates the relative number of voxels selected in V1 for that subject. † marks indicate specific ROIs in specific subjects with low signal quality (and thus few voxels selected for analysis). See Figure S01 for evaluation of signal across subjects. Each horizontal stripe through the image shows the weights for a different voxel. Voxels are sorted within each subject by normalized prediction accuracy for the Fourier power model. Weights from the model that produced the most accurate predictions in V1 are at the top. The solid white line across the image for each subject shows the chance threshold for prediction accuracy (*p* < 0.05, FDR corrected). The bar graph at the top of the panel shows the mean β weights for all V1 voxels for all subjects. Each text label corresponds to both the bar above it and the column of weights below it. Error bars are 99% confidence intervals across all voxels. These tuning patterns are consistent with known response properties of V1, where voxel responses are related to the amount of Fourier power in each image. **(B)** Same plots as **(A)**, for the subjective distance model in V1. Voxels are sorted by normalized prediction accuracy for the subjective distance model. **(C)** Same plots as **(A)**, for the object category model in V1. Voxels are sorted by normalized prediction accuracy for the object category model. **(D–F)** Same plots as **(A–C)**, but for FFA. These tuning patterns are consistent with known response properties of FFA, where voxel responses are related to object categories associated with animate entities.

**Figure 5:**
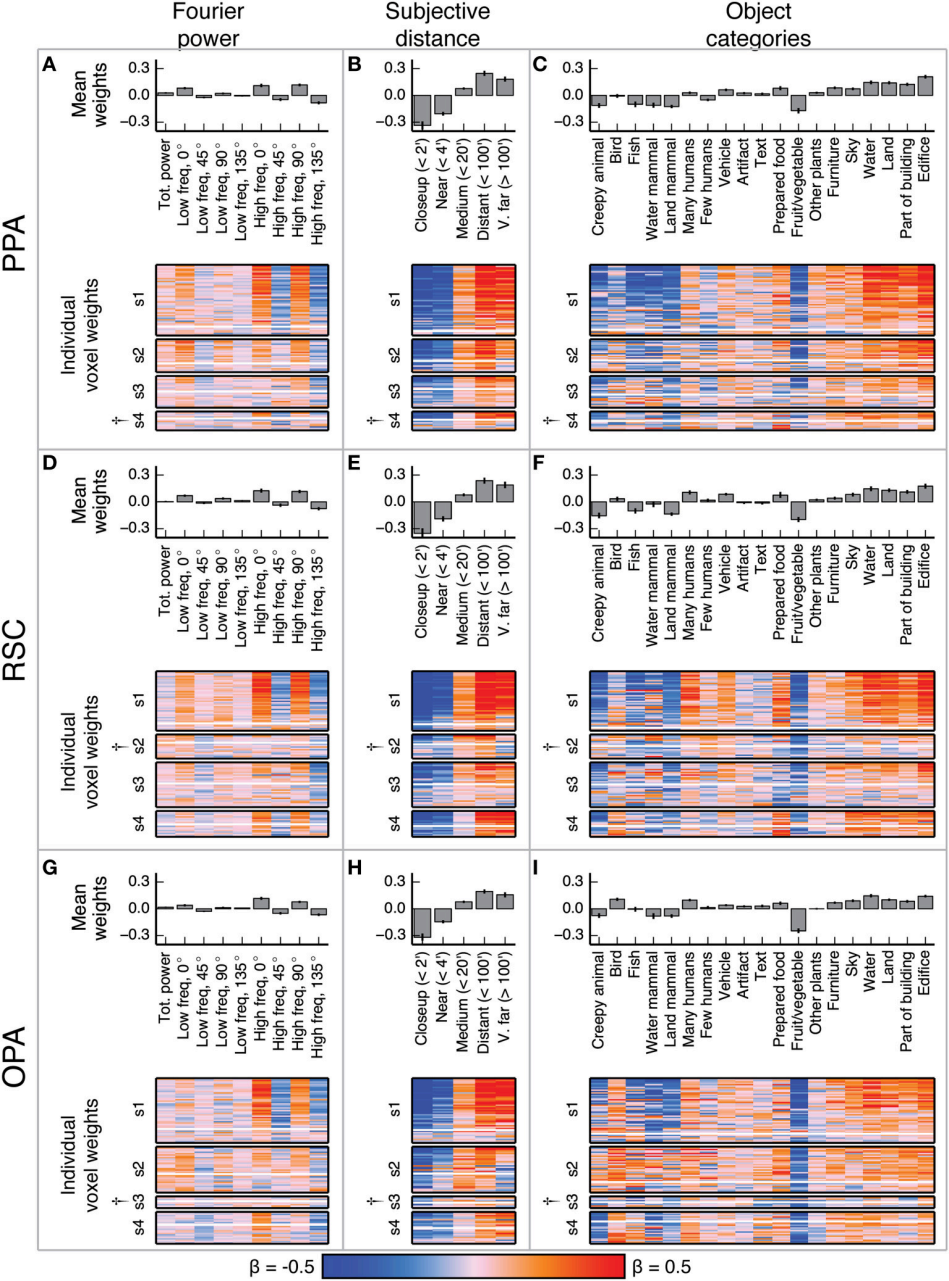
**Voxel-wise model β weights for all models for all voxels in PPA, RSC, and OPA**. **(A–C)** Same plots as Figures 4A–C but for PPA, with conventions as in Figure 4. **(D–F)** Same plots as Figures 4A–C but for RSC. **(G–I)** Same plots as Figures 4A–C but for OPA. † marks indicate specific ROIs in specific subjects with low signal quality (and thus few voxels selected for analysis). See Figure S01 for evaluation of signal across subjects. For the Fourier power model, the voxel-wise β weights are generally large for high frequency cardinal (vertical and horizontal) orientations, though this varies across subjects. For the subjective distance model, voxel-wise β weights are large for distant objects and small for nearby objects across all subjects. For the object category model, voxel-wise β weights were large for object categories related to the scene structure (e.g., *edifice, land*, and *sky*) and small for object categories associated with animate entities (e.g., *few people, land mammal*, and *water mammal*). This pattern of β weights in the object category model was consistent across subjects and ROIs with good signal. All these results are generally consistent with previous reports.

The voxel-wise model β weights for the features in each model are shown in Figures 4, 5. For each area, all voxels for each subject that met our voxel selection criterion [γ > 0.04, *p*(γ) < 0.01—see Methods] are shown. Overall, the tuning profiles revealed by the β weights in each area appear to be broadly consistent with tuning revealed by previous studies. We first describe the β weights in two comparably well-understood areas (V1 and FFA), and then describe the β weights for each model for all three scene-selective areas.

In V1, the β weights for the Fourier power model (Figures 4A–C) show that images containing high Fourier power tend to elicit responses above the mean. This is consistent with many studies showing that V1 responses increase with increasing image contrast (Albrecht and Hamilton, 1982; Gardner et al., 2005). The β weights for the subjective distance model show that very distant scenes elicit responses below the mean in most V1 voxels. This is likely because the most distant scenes (such as the image of the ocean in Figure 1A) have low overall Fourier power. The β weights for the object category model show that the images with labels for *fruit and vegetable, prepared food*, and *creepy animal* all elicit responses above the mean. These are also likely be related to different levels of Fourier power. We analyze the correlations between Fourier power and specific object categories, as well as other correlations between feature channels in different models, in detail below.

In FFA, the β weights for the Fourier power model (Figures 4D–F) show that images with high frequency energy at 135° tended to elicit BOLD responses above the mean, while high frequency energy at vertical and horizontal (90° and 0°) orientations elicit responses below the mean. Several previous studies have rigorously argued that FFA responds to faces rather than low-level image features (Kanwisher and Yovel, 2006). Thus, the tuning for specific frequencies and orientations is likely to reflect natural correlations between the presence of humans or other animate entities and particular spatial frequency patterns. The β weights for the subjective distance model show that relatively nearby objects elicit BOLD responses above the mean in FFA, while distant objects elicit responses below the mean, and the nearest objects do not affect responses in either direction. This is consistent with at least one study that showed parametrically increasing responses in FFA to scenes with increasingly nearby objects (Park et al., 2015). Finally, the β weights for the object category model show that images containing object categories relating to humans and animals elicited BOLD responses above the mean, while images containing categories related to structural features of scenes (*water, land, edifice*, etc.) elicit BOLD responses below the mean. These results replicate well-established tuning properties of FFA (Kanwisher et al., 1997; Kanwisher and Yovel, 2006; Huth et al., 2012; Naselaris et al., 2012), and are consistent across subjects in voxels that have sufficient signal to model (See Figure S01 for assessment of signal quality by subject and ROI).

Figure 5 shows the model β weights for all models and all voxels in PPA, RSC, and OPA. Since the β weights in each of the three models show similar tuning in all three areas, we describe the tuning model by model in all three areas.

The β weights for the Fourier power model (Figures 5A,D,G) show a somewhat variable pattern across subjects. In general, Fourier power at cardinal orientations tends to elicit BOLD responses above the mean in voxels in PPA, RSC, and OPA, while Fourier power at oblique orientations elicits BOLD responses that are small or below the mean. This result is obvious in subject 1, but weaker in the other subjects. In subject 1, the β weights are large for high frequency Fourier power and small for low frequency Fourier power, but this pattern also is weak in the other subjects. We note that subject 1 had substantially better signal (a higher average noise ceiling and more voxels retained) than the other subjects (Figure S01). Thus, the slightly inconsistent tuning across subjects may have been a result of differences in signal quality. The pattern of responses we observe in subject 1 and in the highest-signal voxels in the other subjects are qualitatively consistent with the results of Nasr and Tootell (2012), who found reliably larger responses to cardinal orientations vs. oblique orientations in PPA (Note that in the Nasr and Tootell study, some of the individual voxels within RSC and OPA also showed a cardinal > oblique orientation effect, even though the ROIs as a whole did not).

The β weights for the subjective distance model (Figures 5B,E,H) show that images with distant salient objects elicited BOLD responses above the mean in most voxels in PPA, RSC, and OPA. Images that contain nearby salient objects elicit BOLD responses below the mean in these same areas. These results were consistent across subjects. Several other studies have also found increased responses to distant scenes (vs. nearby scenes) in scene-selective areas (Amit et al., 2012; Park et al., 2015).

The β weights for the object category model (Figures 5C,F,I) show that images containing buildings or vistas (i.e., images with *edifice, water*, and/or *land* labels) elicit BOLD responses above the mean in PPA, RSC, and OPA. Some voxels also respond above the mean to images with *sky* and *furniture* labels. In contrast, images labeled with animate categories (e.g., *land mammal, water mammal*, and *few humans*) elicited BOLD responses below the mean. These results were consistent across subjects. The low weight for the *fruit and vegetable* category is likely due to a bias in stimulus sampling. The stimulus set contained numerous close-up images of fruits and vegetables, such as the top image in Figure 1A. The overall pattern of responses in all three areas is consistent with numerous previous studies that have demonstrated increased responses to landscapes, buildings, and other large, inanimate objects in scene-selective areas (Epstein and Kanwisher, 1998; Huth et al., 2012; Naselaris et al., 2012).

To visualize the cortical extent of each of these patterns of tuning independent of ROIs, we computed three different *t* contrasts between the β weights in each of the models for each voxel in the cortex. We used the β weights from the Fourier power model, the subjective distance model, and the object category model, respectively, to compute contrasts of cardinal vs. oblique, far vs. near, and humans vs. buildings. Each of these contrasts has been emphasized in previous work. Thus, we provide them here for purposes of comparison with other studies that have computed similar maps. However, note that these contrasts are simplifications of the full tuning profile revealed by the weights, particularly for the object category model, which contains many categories besides humans and buildings.

**Figure 6:**
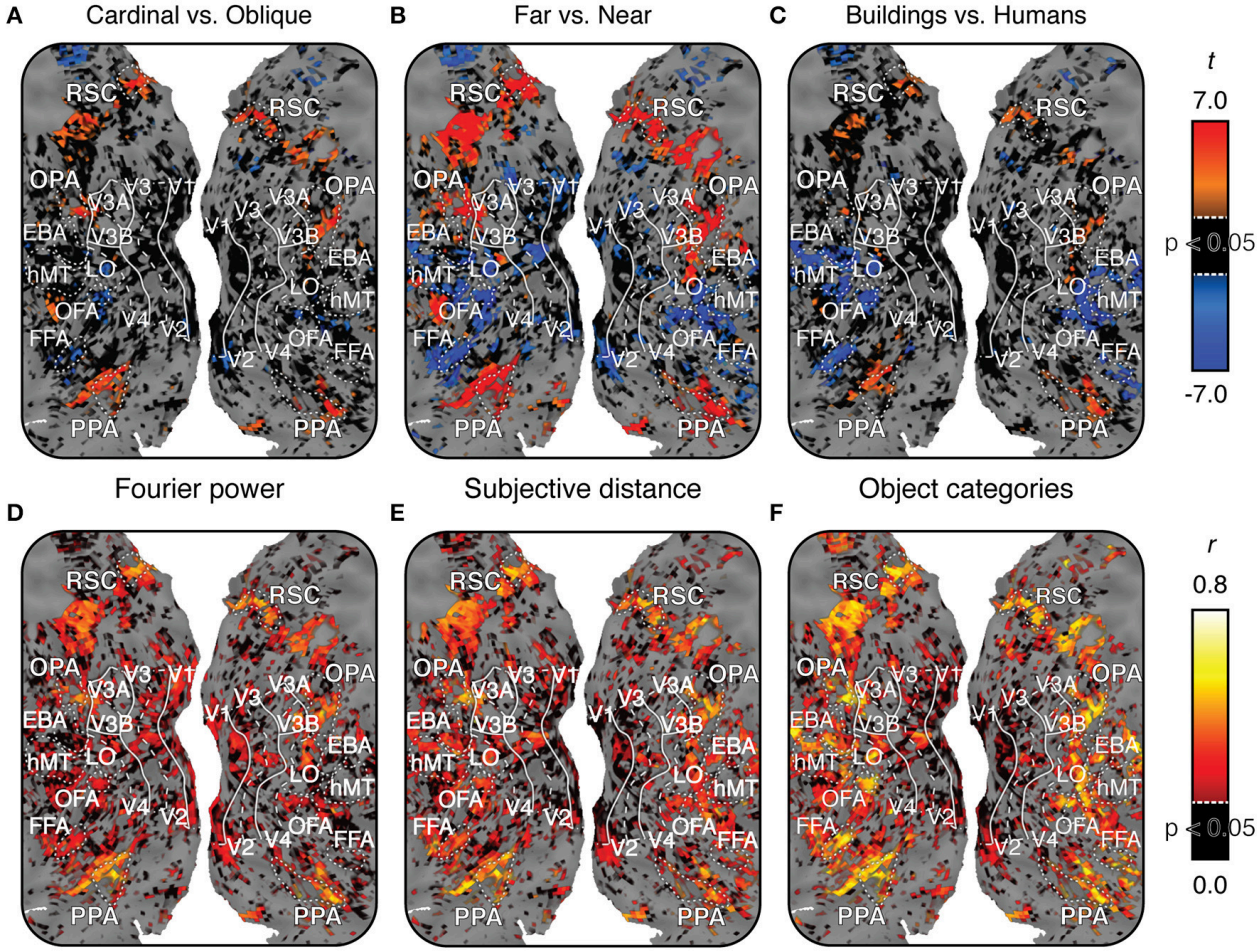
**Maps of voxel-wise *t* contrasts and normalized prediction accuracy for subject 1**. Figures S04–S06 show the same maps for the other three subjects. For all maps, dashed lines indicate the horizontal meridian in the visual field, solid lines indicate the vertical meridian, and dotted lines indicate the boundaries of regions of interest defined by functional contrasts. **(A)** *t* contrast computed for β weights within the Fourier power model (cardinal vs. oblique). *t*-values are scaled from -7 to 7, black voxels indicate *t*-values below the chance threshold (*t* < 3.36, FDR-corrected *p*>0.05) despite good signal [γ > 0.04, *p*(γ) < 0.01]. Gray voxels indicate poor signal [γ < 0.04, *p*(γ) > 0.01] and thus no basis for comparing models. **(B)** *t* contrast for β weights within the subjective distance model (far vs. near). **(C)** *t* contrast computed for β weights within the object category model (buildings vs. people). Voxels with significant *t* contrasts for each of the three models are located in the same regions of the cortex. **(D)** Prediction accuracy for the Fourier power model. Prediction accuracy has been normalized by the noise ceiling. Black voxels indicate correlations that are below the chance threshold (*r* < 0.21, FDR-corrected *p* > 0.05) despite good signal [γ > 0.04, *p*(γ) < 0.01]. Gray voxels indicate poor signal [γ < 0.04, *p*(γ) > 0.01], and thus no potential to test predictions. **(E)** Prediction accuracy for the subjective distance model. **(F)** Prediction accuracy for the object category model. All three models make accurate predictions in similar locations across the cortex, though the object category model makes more accurate predictions in FFA, OFA, and EBA. Combined with the *t* contrast maps, this suggests that the three different models may each describe the same response variance in scene-selective areas in a different way.

Figures 6A–C show each of these contrasts for one subject, projected onto that subject's cortical surface. Figures S04–S06 show the same maps for the other three subjects. For all three contrasts, many voxels with reliably large (*p* < 0.05, FDR corrected) positive *t*-values are located in PPA, RSC, and OPA. Relatively few voxels outside scene-selective areas have large positive *t*-values (Some voxels in the posterior medial parietal lobe also show large *t*-values in some subjects, particularly for the near vs. far contrast). These contrasts are broadly consistent with contrast maps reported in other studies (Rajimehr et al., 2011; Amit et al., 2012; Nasr and Tootell, 2012; Park et al., 2015). However, as in Figure 5, there is variability across subjects in the weights in the Fourier power model. Thus, our replication of tuning for cardinal orientations (as observed by Nasr and Tootell, 2012) is weaker than our replication of tuning for far distances and categories associated with scene structure.

In summary, the voxel-wise models of Fourier power, subjective distance, and object categories reveal three qualitatively different patterns of tuning that are common to all three scene-selective areas: (somewhat) stronger responses to cardinal than to oblique orientations, stronger responses to distant than to nearby objects, and stronger responses to object categories associated with buildings and landscapes than to categories associated with animate objects. However, the tuning revealed by the voxel-wise model β weights does not reveal which of the three models provides the best overall account of the responses in each area. Furthermore, some of the tuning results in V1 and FFA suggest that correlations between features in different models may have affected the estimated tuning for each model (For example, it seems unlikely that V1 truly represents fruits and vegetables, as Figure 4 seems to indicate). We address both of these issues below.

### The object category model makes the best predictions in scene-selective areas

To determine which model provides the best description of BOLD responses in each area, we used each fit model to predict responses in a separate validation data set (Figure 1). We then computed the correlation between the predictions of each model and the estimated BOLD responses in the validation data. Correlations were normalized by the estimated noise ceiling for each voxel.

Figures 6D–F show estimates of prediction accuracy for all three models for one subject projected onto that subject's cortical surface. Figures S04–S06 show similar maps for the other three subjects. All three models accurately predict brain activity in PPA, RSC, and OPA. The object category model also makes good predictions in the FFA, the Occipital Face Area (OFA), and the Extrastriate Body Area (EBA), as reported previously (Naselaris et al., 2012). This is likely because the object model contains labels for the presence of humans and other animate categories.

**Figure 7:**
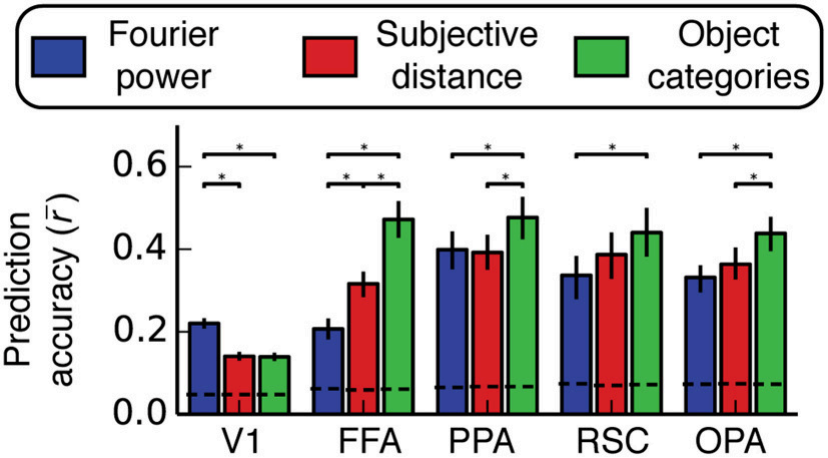
**Prediction accuracy (Pearson's *r*) averaged across all voxels and all subjects within several different regions of interest**. Predictions are normalized by the noise ceiling and only voxels with reliable stimulus-evoked responses are included. Error bars are 99% confidence intervals, asterisks indicate significant differences between models (bootstrapped *p* < 0.05), and the dotted lines across the bottom indicate the chance threshold (bootstrapped *p* = 0.05) for the mean correlation for each ROI (Thresholds differ slightly across ROIs because of the differing number of voxels in each ROI). The Fourier power model makes the best predictions in V1, and the semantic category model makes the best predictions in all other ROIs (except in RSC, where the subjective distance model and the semantic category model are not reliably distinguishable). We note, however, that the object category model was not reliably better than the Fourier power and subjective distance models in all three scene-selective areas in all subjects (see Figure S07 for individual subject results).

Figure 7 shows estimates of prediction accuracy for all three models, averaged across voxels in all four subjects within each of several different ROIs. Figure S07 shows the same result for each individual subject.

In area V1 the Fourier power model provides the best predictions of brain activity (bootstrap *p* < 0.05). This suggests that tuning in the Fourier power model (Figure 4A) is more important than tuning in the subjective distance and object category models in V1 (Figures 4B,C). In FFA the object category model provides the best predictions (all bootstrap *p* < 0.05). This suggests that tuning in the object category model (Figure 4F) is more important than tuning in the Fourier power or subjective distance models in FFA (Figures 4D,E). Thus, in both V1 and FFA, choosing the best model based on prediction accuracy favors the models that are most consistent with previous results for these areas (Jones and Palmer, 1987; Kanwisher and Yovel, 2006; Kay et al., 2008; Naselaris et al., 2009). These examples demonstrate how assessing prediction accuracy can (and should) affect the interpretation of tuning revealed by β weights.

In PPA, the object category model provides the best predictions of brain activity (all bootstrap *p* < 0.05). This suggests that tuning in the object category model is more important than tuning in the Fourier power or subjective distance models in PPA. In RSC, the object category model provides more accurate predictions than those provided by the Fourier power model (bootstrap *p* < 0.05), but the predictions of the object category model are not significantly different from those of the subjective distance model (bootstrap *p* = 0.14). This suggests that tuning in the object category model is more important than tuning in the Fourier power model, but it is unclear whether the tuning in the subjective distance model or the tuning in the object category model is more important. In OPA, the object category model provides the best predictions of brain activity (all bootstrap *p* < 0.05). Thus, as in PPA, tuning in the object category model is more important than tuning in the Fourier power or subjective distance models in OPA.

Among the options tested here, the representation in two of three scene-selective areas (PPA and OPA) is best described in terms of tuning for object categories. In RSC, tuning for object categories is more important than tuning for Fourier power. Thus, the object category model seems to be a good model for all three areas. However, this conclusion is weakened by variability in relative prediction accuracy across individual subjects (Figure S07). Furthermore, the fact that all three models make quite accurate predictions in all three areas (across all subjects with good signal) suggests that each model may each describe the same underlying representation in different ways.

### The fourier power, subjective distance, and object category models all explain the same response variance

The Fourier power, subjective distance and object category models all provide accurate predictions of BOLD responses in scene-selective visual areas. Given this result, an obvious question arises: do the Fourier power and subjective distance models explain the same BOLD response variance as is explained by the object category model? That is, can tuning for Fourier power and/or subjective distance almost fully account for category tuning? This question cannot be answered by merely examining prediction accuracy, because two models that make comparably accurate predictions could describe either unique or shared components of response variance (see example in Figure 3B). We performed a variance partitioning analysis to determine whether the three models explain unique or shared response variance in the ROIs of interest here. Variance partitioning allocates variance to each model based on whether two models can be combined for a gain in variance explained. If they can, then each model explains unique response variance; if not, the variance explained by the models is shared (see Figure 3 and Methods for an overview).

**Figure 8:**
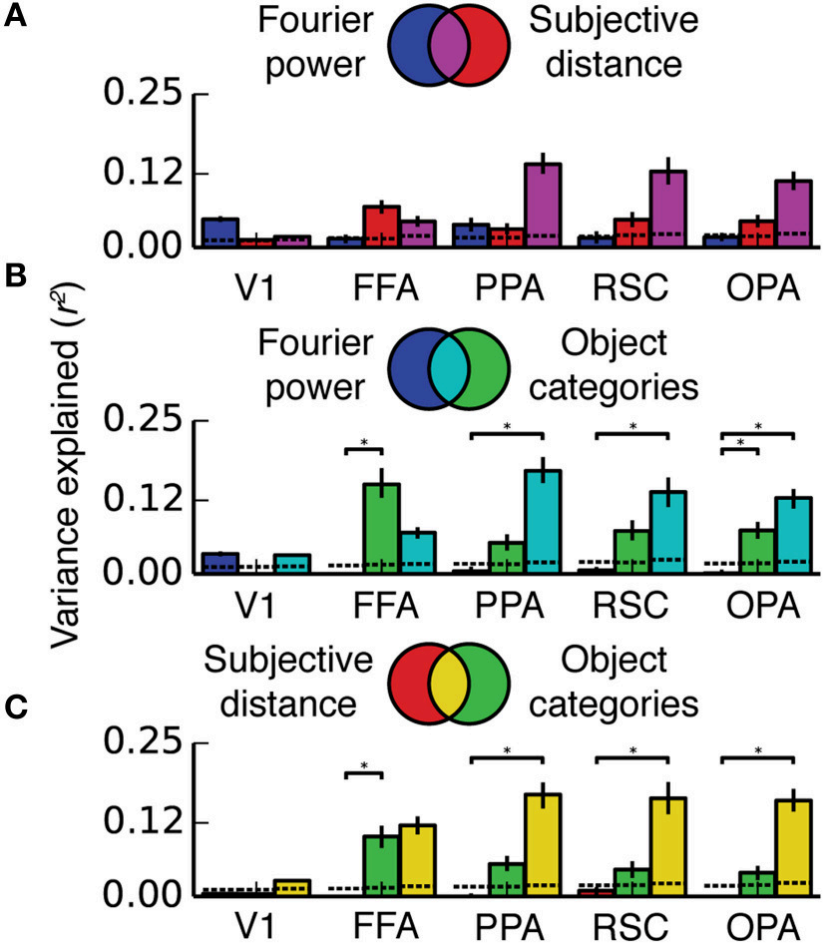
**Two-way variance partitioning analyses**. All plots are based on concatenated data for all four subjects. **(A)** Independent and shared variance explained by Fourier power and subjective distance models. Dotted lines at the bottom of the graph indicate chance levels (bootstrapped *p* = 0.05) of variance explained, and asterisks indicate significant differences in variance explained (bootstrapped *p* < 0.05). Error bars are 99% confidence intervals across all voxels in a region. **(B)** Independent and shared variance explained by Fourier power and object category models. **(C)** Independent and shared variance explained by subjective distance and object category models. In PPA, RSC, and OPA, all pairs of models share a substantial amount of variance. Compared to the object category model, neither the Fourier power model nor the subjective distance model explains any unique variance.

**Figure 9:**
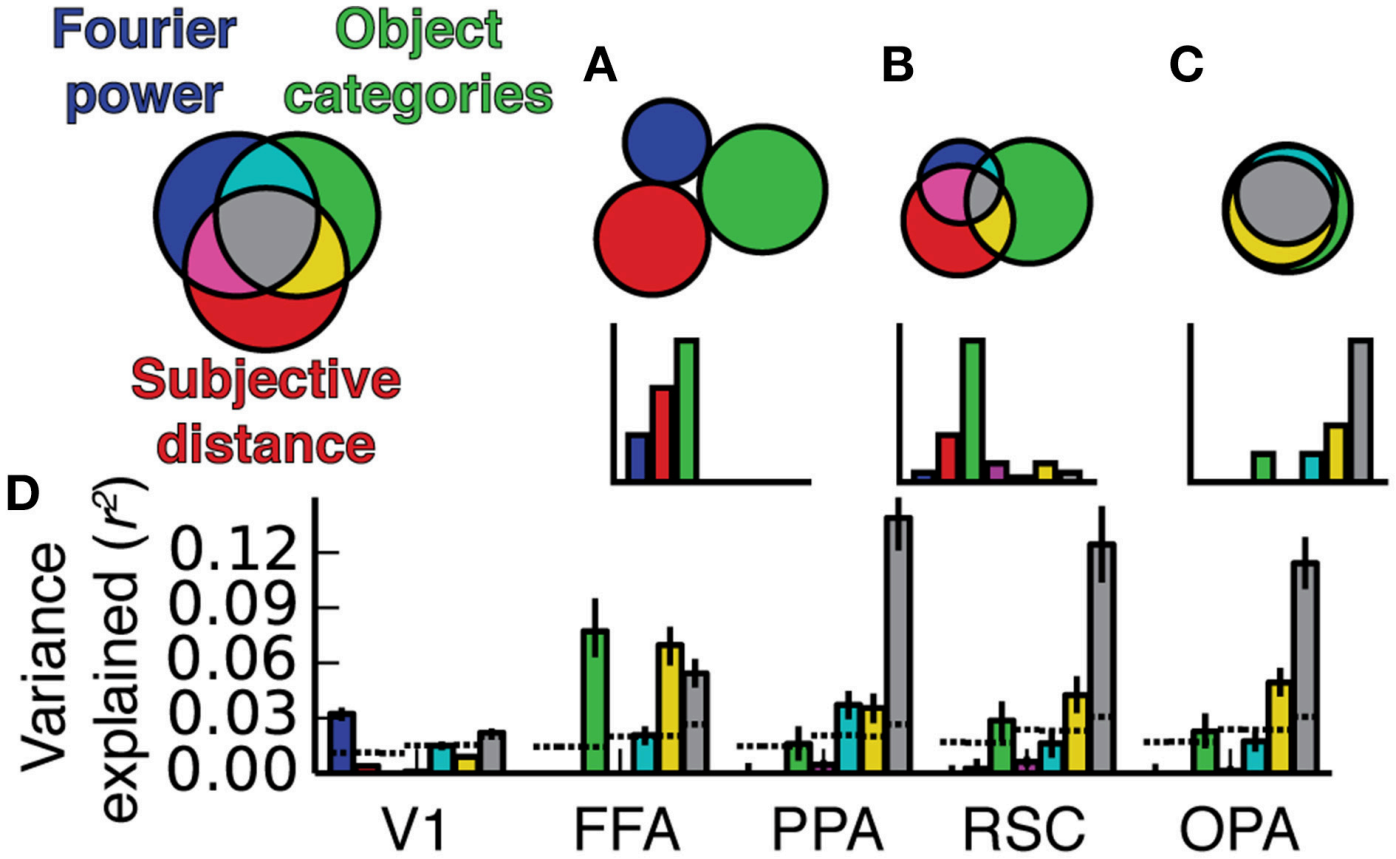
**Three-way variance partitioning analysis**. **(A)** Venn diagram representing hypothetical relationships between the variance explained by the three models, such that each explains a unique component of the variance. **(B)** A different hypothetical relationship in which the semantic category model explains a large fraction amount of independent variance, but the three models all share small amounts of variance. **(C)** A third possible relationship in which all three models explain shared variance, and the Fourier power and subjective distance models account for most of the variance explained by the object category model. **(D)** Three-way variance partitioning results obtained in our experiment. This plot is based on concatenated data for all four subjects; results for individual subjects are shown in Figure S08. Dotted lines at the bottom of the graph indicate chance thresholds (bootstrapped *p* = 0.05) for the amount of variance explained. Chance thresholds differ depending on the number of voxels per ROI and the number of subtractions between fit models necessary to compute each component of the variance. Error bars are 99% confidence intervals across all voxels in a region. The pattern of results is most consistent with the Venn diagram in **(C)**.

Figures 8, 9 show the results of the variance partitioning analysis. In V1, only the Fourier power model explains any unique variance that cannot be explained by the other two models. All three models also share a small amount of variance in V1. The shared variance is likely due to natural correlations between specific features that affect responses in V1 and other features. For example, images with distant objects often have low overall contrast (and thus low Fourier power, as the image of the ocean in Figure 1A); thus distance and Fourier power are likely to be correlated (We analyze correlations between all features in detail below). Since total Fourier power affects responses in V1 (Figure 4A), this correlation could lead to the subjective distance model and the Fourier power model providing similar predictions (and thus explaining shared variance). Thus, it is likely that the subjective distance and object category models only explain any variance in V1 (Figure 7) because of the variance that they share with the Fourier power model.

In FFA, only the object category model explains any unique variance. All three models also share a significant amount of variance, and the subjective distance model and the object category model share a significant amount of variance that is independent of the Fourier power model. The unique variance explained by the object category model is in keeping with known response properties of FFA (Kanwisher and Yovel, 2006; Huth et al., 2012; Naselaris et al., 2012). As in V1, the shared variance between the object category model and the subjective distance model may be due to natural correlations between features. For example, people and other animate categories are more likely to be present at specific distances (in this particular stimulus set, and also potentially in natural visual experience in general). Interestingly, at least one other study has found similar tuning for distance in FFA (Park et al., 2015). However, this study may be subject to the same stimulus feature correlations.

In scene-selective areas PPA, RSC and OPA, most of the variance explained by the Fourier power, subjective distance, and object category models is shared among all three models (Figure 9). That is, most of the variance explained by any one of the three models is explained by all three models. Only the object category model explains any unique variance in PPA, RSC, or OPA that cannot be explained by the other two models (Figure 9). Thus, the Fourier power and subjective distance models provide partial (but not complete) explanations of variance explained by the object category model in scene-selective areas.

The Fourier power and subjective distance models could be favored on grounds of parsimony, since both models have fewer feature channels than the object category model, and both Fourier power and distance are presumably less complex to compute than abstract category labels. However, neither simpler model provides a more accurate description of BOLD responses in scene-selective areas than that provided by the object category model, and neither model predicts any variance that is not already accounted for by the object category model.

### Fourier power, subjective distance, and object category labels are highly correlated in natural images

**Figure 10:**
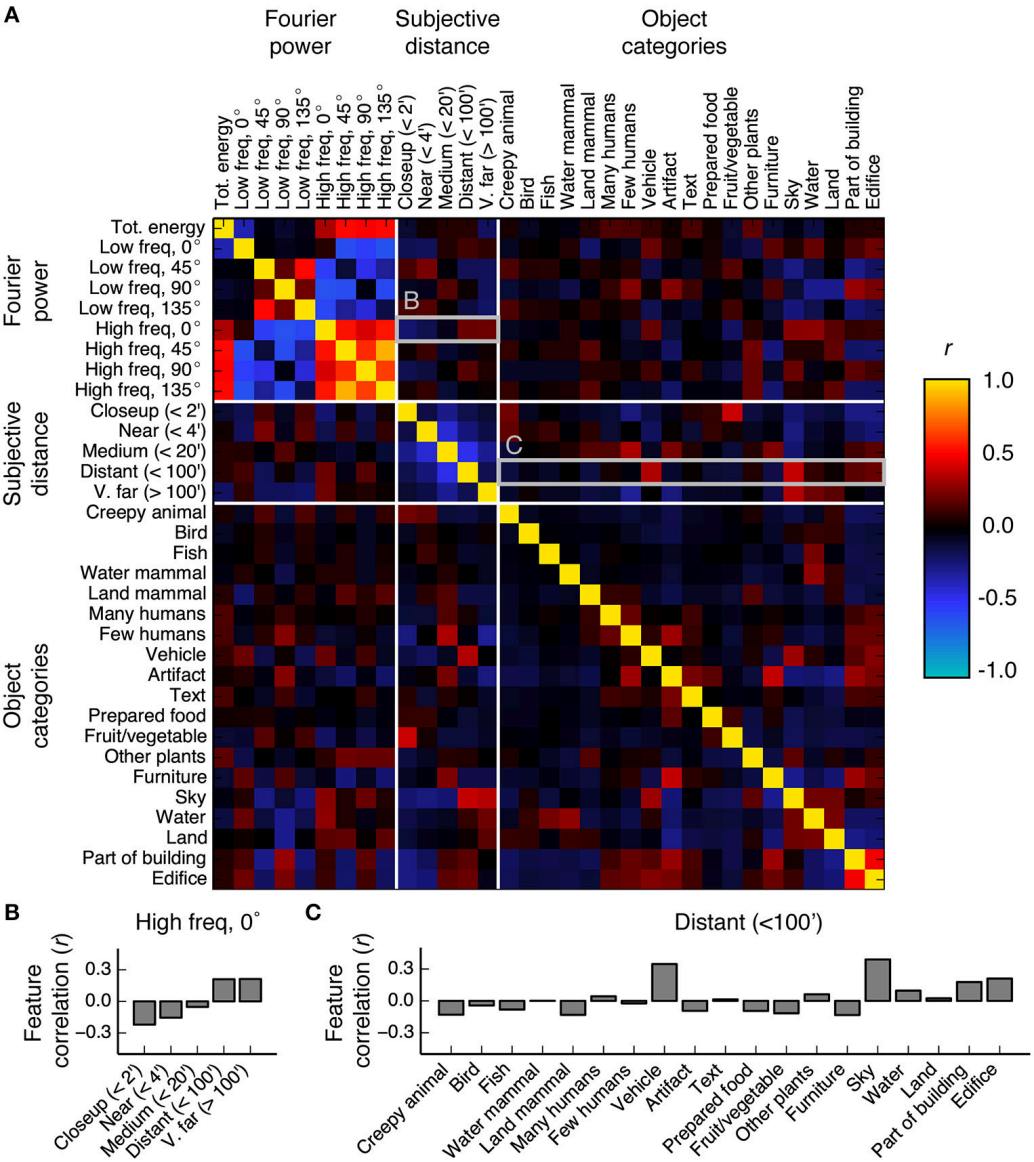
**Correlations between all features in the Fourier power, subjective distance, and object category feature spaces**. **(A)** Full correlation matrix. White lines demarcate boundaries between feature spaces. Features that elicit responses above the mean in scene-selective areas [the Fourier power features labeled *High freq*, 0° and *High freq*, 90°; the subjective distance features labeled *Distant (*<*100*′*)* and *V. far (*>*100*′*)*; and the semantic category labels *Edifice, Part of building, Water, Land*, and *Sky*] tend to have high correlations between them. Panels **(B,C)** provide zoomed in views of the correlation values for the rows marked **(B,C)** in the correlation matrix. **(B)** Bar graph of the correlations between the Fourier power channel *High freq*, 0° and all subjective distance features. High frequency horizontal Fourier power is positively correlated with large subjective distances, potentially due to the presence of a thin horizon line and tiny objects in faraway scenes. **(C)** Bar graph of the correlations between the subjective distance channel *Distant (*<*100*′*)* and all object category features. Distant scenes are tend to have the labels *Vehicle, Sky, Part of building*, and *Edifice*. The high correlations between features with high β weights in scene-selective areas could be a consequence of all three models attempting to parameterize the space of scene features.

The shared variance among the three models in PPA, RSC, and OPA is likely due to correlations between features in the feature spaces underlying the models. To investigate this possibility we computed the correlations between all features in the Fourier power, subjective distance, and object category feature spaces. Figure 10 shows the resulting correlations. The highest correlations are between the features within the Fourier power feature space. This was expected, since correlations between different spatial frequency bands are a well-known property of natural images (Field, 1987). The average correlation magnitude for features in different feature spaces is *r* = 0.11.

We found reliable relationships between several Fourier power and subjective distance channels. For example, Figure 10B shows that horizontal high frequency Fourier power is positively correlated with far distances and negatively correlated with near distances. These correlations may be a result of thin horizontal horizon lines in distant images. Conversely, two low frequency Fourier power channels (*Low freq* 45° and *Low freq* 135°) are positively correlated with near and medium distances and negatively correlated with far distances. Vertical low frequency Fourier power is also positively correlated with intermediate distances and negatively correlated with far distances. The correlations between most low frequency channels and near distances could be a result of perspective projection: nearby objects will fill more of the visual field, and thereby increase low frequency Fourier power. Low frequency horizontal Fourier power may not follow the same trend as other low frequency orientations because the land/sky boundaries will increase both high and low horizontal Fourier power in distant scenes.

**Figure 11:**
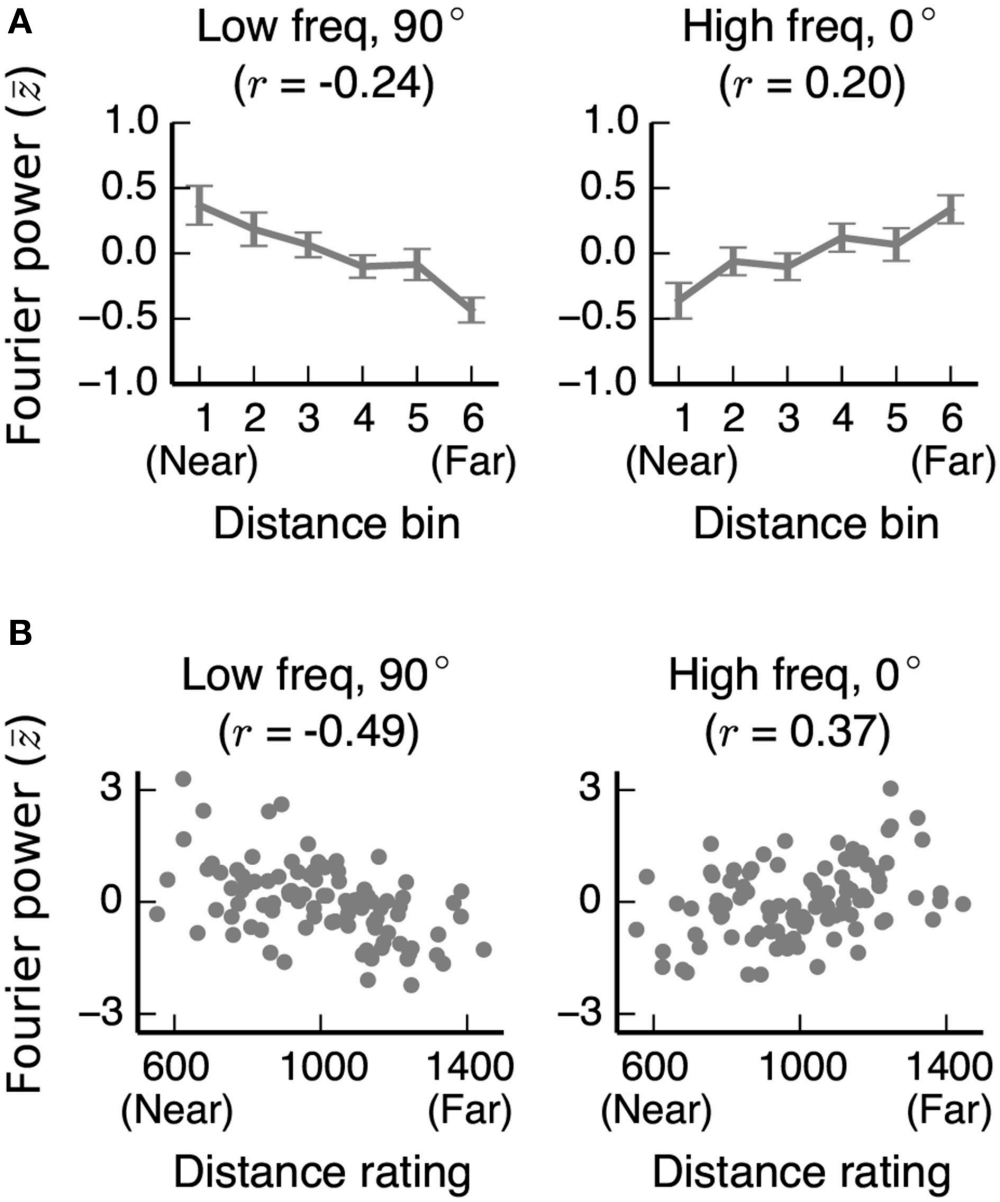
**Correlations between distance and two Fourier power channels in stimulus sets from other studies**. **(A)** Mean low frequency vertical and high frequency horizontal Fourier power for each distance bin for images used in Experiment 2 of Park et al. (2015). Fourier power channels were *z*-scored across all images in the stimulus set before averaging across bins. Error bars are standard errors of the mean. **(B)** Low frequency vertical and high frequency horizontal Fourier power for each image in Kravitz et al. (2011), plotted against the behavioral distance ratings for each image obtained in that study. In both stimulus sets, as in our stimulus set, low frequency vertical (90°) Fourier power is reliably associated with nearer scenes, and high frequency horizontal (0°) Fourier power is reliably associated with far-away scenes.

To determine whether the relationships between Fourier power and distance that we observe are general to other stimulus sets as well, we computed the same Fourier power feature space for the stimuli used in two previous fMRI studies of distance representation (Kravitz et al., 2011; Park et al., 2015). In both stimulus sets, we found the same relationships between Fourier power and distance as in our stimuli (Figure 11; See Figures S09, S10 for further analysis of these two data sets).

We also note that many of the features that elicit large responses in scene-selective areas (Figure 5) have relatively high correlations with each other. For example, the category label *sky* is correlated with the subjective distance label *Distant (*<*100*′*)* (*r* = 0.39), and horizontal high-frequency Fourier power (Fourier power channel *High freq*, 0°) is correlated with the semantic labels *vehicle, sky*, and *water* (*r* = 0.19, 0.27, and 0.27, respectively). Each of these labels is fairly common in the stimulus set (each occurs in at least 230/1326 images—see Figure S03 for frequencies of all object category and distance labels). Thus, the correlations between Fourier power feature channels and the category labels *vehicle, sky*, and *water* are reasonably likely to be representative of natural relationships between features in the real world.

Other correlations between less common labels may reflect sampling biases in this particular set of images. For example, the correlation between the nearest distance label [*Closeup (*<*2*′*)*] and the object label *Fruit/vegetable* is 0.39. *Fruit/vegetable* only occurs in 62 images, of which 32 are rated as *Closeup (*<*2*′*)*. The relative rarity of the *Fruit/vegetable* labels, combined with the observation that fruits do not usually appear less than two feet from one's face, suggest that this correlation is potentially spurious.

Whether feature correlations are due to natural statistics or sampling biases, there is a risk that they will lead to biases in estimation of weights, and thereby to models that spuriously share variance. However, it is unclear whether correlations of the magnitude that we observe will necessarily give rise to models that share variance.

### A combination of correlations between features and voxel tuning produce shared variance

We performed a simulation to illustrate how the feature correlations and voxel-wise β weights in our experiment give rise to models that explain the same variance. We generated two simulated data sets. The first was based on the stimulus feature spaces and the β weights estimated from the fMRI data for voxels in scene-selective areas, and the other was based on the same feature spaces and a set of semi-random β weights (see Methods for details). The two sets of β weights differed in whether the features that were correlated across feature spaces had relatively high β weights or not (the real weights did, but the random weights generally did not). We applied the same variance partitioning analysis that we previously applied to the fMRI data to both sets of simulated data.

**Figure 12:**
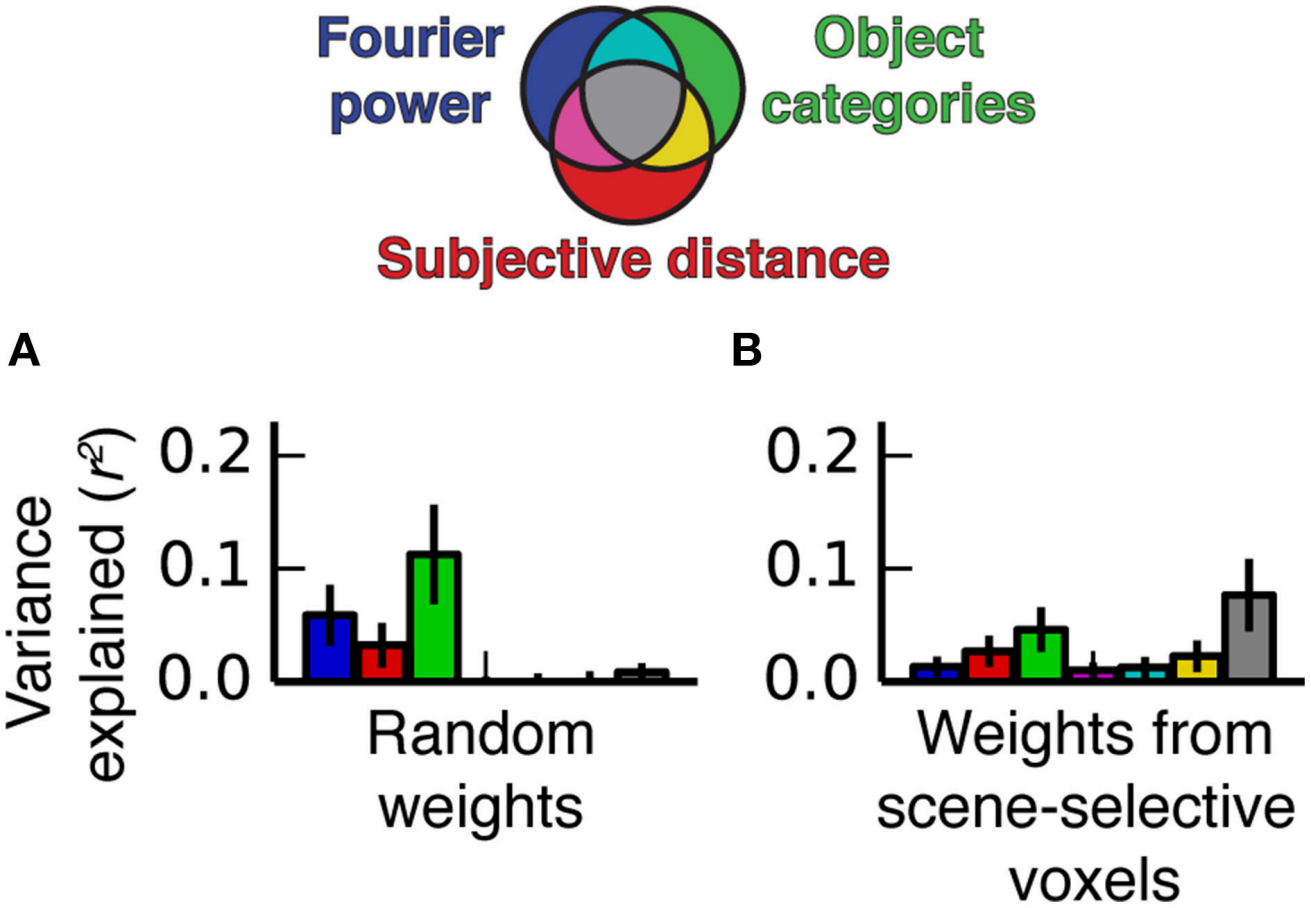
**Simulated variance partitioning. (A)** Variance partitioning conducted on simulated data generated based on the feature spaces for all three models and a set of semi-random β weights (see Methods for details). This shows that, despite the correlations between feature spaces, there are many patterns of tuning that could result in estimates of unique variance explained for each model. **(B)** Variance partitioning conducted on simulated data generated based on the feature spaces for all three models and actual β weights from voxels in scene-selective areas. This shows that the specific pattern of tuning that we observed (with high weights on the most correlated features) is likely to result in shared variance across these three models.

Figure 12 shows the results of the simulation. When semi-random β weights were used to generate the simulated data, the variance partitioning still detected unique variance explained by each model despite the correlations between some of the features in the feature spaces. However, when the real β weights were used to generate the simulated data, the variance partitioning analysis found a large fraction of shared variance between all three models. Thus, the simulation makes it clear that correlated features in different feature spaces only lead to shared variance if the correlated features also have relatively high β weights. The β weights, which reflect the specific response properties of PPA, RSC, and OPA, can selectively magnify correlations between particular correlated features when predictions are computed, which can lead to shared variance between the different models.

This suggests that new models of scene-selective areas are more likely to explain unique variance to the extent that the features they parameterize are *not* correlated with other features known to be associated with responses in scene-selective areas.

## Discussion

Several areas in the human brain respond to visual scenes, but which specific scene-related features are represented in these areas remains unclear. We investigated three hypotheses that have been proposed to account for responses in scene-selective areas such as PPA, RSC, and OPA. Specifically, we investigated whether these areas represent (1) information about the Fourier power of scenes, (2) the subjective distance to salient objects in scenes, or (3) semantic categories of scenes and their constituent objects. We evaluated these three hypotheses by applying voxel-wise modeling to a data set consisting of BOLD fMRI responses elicited by a large set of natural images. We created and compared the prediction performance of three voxel-wise encoding models, one reflecting each of these alternative hypotheses.

We found that a voxel-wise model based on semantic categories makes slightly more accurate predictions than a model based on Fourier power (in PPA, RSC, and OPA) or subjective distance (in PPA and OPA). However, a variance partitioning analysis revealed that, in all three areas, the variance predicted by these three models is mostly shared. The shared variance is likely a result of a combination of the response patterns of voxels in scene-selective areas and high natural correlations between the stimulus features in the feature spaces underlying each of the models. We therefore conclude that any or all of these models can provide a plausible account of visual representation in PPA, RSC, and OPA.

### Previous studies have not resolved which model best describes scene-selective areas

Several previous studies of PPA, RSC, and/or OPA have argued in favor of each of the hypotheses tested here, or in favor of closely related hypotheses (Walther et al., 2009; Kravitz et al., 2011; Park et al., 2011, 2015; Rajimehr et al., 2011; Nasr and Tootell, 2012; Watson et al., 2014). However, none have completely resolved which features are most likely to be represented in scene-selective areas. We briefly review three representative and well-designed studies of scene-selective areas here, and assess their conclusions in light of our results.

Nasr and Tootell argued that PPA represents Fourier power (Nasr and Tootell, 2012). Specifically, they showed that filtered natural images with Fourier power at cardinal orientations elicit larger responses in PPA than do filtered images with Fourier power at oblique orientations. In two control experiments, they measured fMRI responses to stimuli consisting of only simple shapes, and found the same pattern of responses. Thus, their results suggest that Fourier power at cardinal orientations influences responses in PPA independent of subjective distance or semantic categories. This in turn suggests that the Fourier power model in our experiment should predict some unique response variance that is independent of the subjective distance and semantic category models. We did find that the Fourier power model gave accurate predictions in scene-selective areas. However, we did not find any unique variance explained by the Fourier power model. There are at least two possible explanations for this discrepancy. First, the Fourier power model may explain some unique variance, but we may have mischaracterized it as shared variance because of stimulus correlations. Second, the results of Nasr and Tootell's study, which relied on filtered and artificial stimuli, simply may not generalize to explain responses to natural images. This is a known pitfall of using artificial or manipulated stimuli (Talebi and Baker, 2012). In any case, the data from the Nasr and Tootell study provide no information about the strength of the relationship between Fourier power and BOLD responses in scene-selective areas relative to the effects of other features. Thus, their study cannot resolve the question of which model is best, nor the question of how Fourier power features are related to other features.

Park et al. (2015) argued that PPA and RSC represent scene size. Their metric for scene size was based on human judgments, and so is closely related to the subjective distance model that we tested here. They measured BOLD responses to a large and carefully chosen set of photographs of natural scenes, and found that responses in PPA and RSC increased parametrically with scene size. However, we found a strong relationship between scene size and Fourier power in the images used in the Park et al. study (Figure 11A, Figure S10). To try to avoid just such confounds, Park and colleagues created a control stimulus set in which high-frequency Fourier power was approximately equalized across different scene sizes. We did not test this control stimulus set directly, but since the differences in Fourier power that we observed were specific to particular orientations, it is unlikely that their control removed all Fourier power differences between scenes. This suggests that differences in particular Fourier power channels between different scene sizes might account for the results reported in the Park study, just as both the Fourier power and subjective distance models provide equivalent descriptions of scene-selective regions in our data. Finally, Park and colleagues did not assess whether the specific semantic categories of objects in each of their scenes might have affected BOLD responses. Without this comparison, it is unclear whether the presence of different object categories in their scenes may have also affected their results. For all these reasons, the results reported by Park and colleagues cannot provide a basis for choosing between the three models of scene-selective areas that we consider.

Kravitz et al. (2011) argue that PPA and OPA represent scene expanse (defined as the difference between open and closed scenes) and relative distance (defined as the difference between near and far scenes). They find that voxel patterns in PPA and OPA distinguish both open scenes from closed scenes and near scenes from far scenes better than the same voxels distinguish natural from manmade scenes. However, variation in Fourier power across their experimental conditions complicates the interpretation of their results. They acknowledge that the open and closed scenes in their stimulus set have visibly different Fourier power spectra. When we processed their stimuli with our Fourier power model, we found significant differences between their open and closed scenes in several Fourier power channels (Figure S09A). This suggests that the different patterns of responses they observed to open and closed scenes could be equally well explained by differences in Fourier power between open and closed scenes. Kravitz et al. do not report any differences between the Fourier spectra of the near and far scenes in their stimulus set. Our analysis of their stimuli also does not find any reliable difference in any Fourier power channel between their near and far scenes (Figure S09B). However, their Near and Far condition labels were based on relative distance within each scene category, which means that the scenes in the Near condition were not necessarily all the subjectively nearest scenes. For example, half their images of beaches were labeled as Near and half their images of hallways were labeled as Far, regardless of whether the beaches were subjectively nearer than the hallways. They did, however, obtain a measure of the relative subjective distance of each scene. When we compared the Fourier power features for each image to these distance ratings (instead of to the near/far condition labels), we found reliable correlations between Fourier power and relative subjective distance in their stimulus set (Figure 11B and Figure S09C), just as in our stimuli and in the Park et al. (2015) stimuli. Thus, the correlation between subjective distance ratings and fMRI-based distance scores reported in Kravitz et al. (2011) might be explained by variation in Fourier power—specifically, by the presence of high frequency horizontal Fourier power in distant scenes. In sum, our reanalysis of the stimuli from Kravitz et al. (2011) suggest that their results cannot provide a basis for choosing between the three models of scene-selective areas that we consider here.

### Other hypotheses regarding scene-selective areas

The Fourier power, subjective distance, and object category feature spaces that we investigated broadly sample the space of hypotheses regarding the representation in scene-selective areas. However, three specific feature spaces obviously do not constitute a comprehensive test of every hypothesis in the literature.

Several other feature spaces have been proposed that parameterize variation in the same three broad domains that our models do (low-level image features, 3D spatial layout, and categorical or semantic information), but with different parameters. For example, low-level image variation can be parameterized using Gabor wavelets (Jones and Palmer, 1987; Kay et al., 2008), scene gist (Oliva and Torralba, 2001; Watson et al., 2014), or extended contours (Walther et al., 2011). 3D spatial variation can be parameterized according to scene expanse (Kravitz et al., 2011; Park et al., 2011) or local scene structure (Epstein and Kanwisher, 1998; Kornblith et al., 2013). And categorical information about scenes can be parameterized using hierarchical object labels (Huth et al., 2012) or labels for categories of scenes rather than objects, including distinctions between natural and man-made scenes (Naselaris et al., 2009; Walther et al., 2009; Stansbury et al., 2013).

Previous studies have also proposed that scene-selective areas may represent scene familiarity (Epstein et al., 2007), landmarks (Janzen and van Turennout, 2004; Auger et al., 2012), or other scene features relevant for navigation (Epstein, 2008; Morgan et al., 2011). None of these hypotheses are obviously related to the feature spaces we investigated.

Any of these feature spaces, if they were formalized and tested in the voxel-wise modeling framework, could potentially yield better or more unique models of BOLD responses than those we tested. However, all these other feature spaces—particularly those in the same broad categories of hypotheses as our models—may be strongly related to each other in the same way that the feature spaces we tested are. Our work provides a blueprint for how to address the correlations between feature spaces in a quantitative and principled way, and to assess which models explain unique or shared variance.

### Suggestions for further studies on representation in scene-selective areas

Our study suggests that the data available currently are not sufficient to discriminate between the alternative hypotheses that scene-selective areas represent information about Fourier power, subjective distance, or object categories. It could be the case that scene-selective areas represent all of these distinct feature classes. Alternatively, it could be the case that scene-selective areas represent only one of these three distinct classes of features, but that the presence of stimulus correlations in our study and missing controls and analyses in previous studies have precluded identification of the most appropriate feature space. Is there any way to resolve this issue?

The only way forward is to test the same models (and/or related models) on different stimulus sets, and to search for stimuli for which some models fail to make accurate predictions of brain responses and other models succeed. However, new stimuli must be chosen carefully to reduce the correlations between stimulus features in different alternative models. Simply removing problematic features (e.g., by Fourier bandpass filtering the stimuli) is not a good solution because the visual system is highly nonlinear (Carandini et al., 2005; Wu et al., 2006). Spatial frequencies that are filtered out of a stimulus may be reintroduced within the visual system by nonlinear processes operating at any level. An analogous process occurs in the missing fundamental phenomenon, which is well known in audition (Wightman, 1973a,b).

Restricting feature variation in experimental stimuli to avoid correlations between features is also not a good solution. This approach might produce satisfying results within the range of stimuli tested in an experiment, but the resulting model will be unlikely to generalize to the larger range of stimuli encountered in the natural world (Talebi and Baker, 2012). This is a lesson that has been well learned in the visual neurophysiology community over the past 20 years: if models are developed using filtered, constrained or highly artificial stimuli, they tend to perform poorly when tested on natural images (David et al., 2004; Talebi and Baker, 2012).

We suggest that one useful way forward would be to create natural stimulus sets that reduce the covariance of stimulus features while maintaining a natural range of variance in as many features as possible. It might be possible to generate stimuli that satisfy these constraints parametrically. Alternatively, it might be possible to develop an appropriate stimulus set by sampling images from an extremely large online database such as ImageNet (http://www.image-net.org/) or the Flickr image database (https://www.flickr.com/creativecommons/). A stimulus set that is designed specifically to minimize covariance between features while maintaining natural variability will reduce the amount of shared variance between models, and lead to clearer conclusions as to which model is best for each area.

Our suggestion that new stimulus sets should be developed is not completely novel. The imperative to include a reasonable amount of natural variation in a stimulus set seems to be an implicit guiding principle in many studies (e.g., Kravitz et al., 2011; Park et al., 2015). However, such implicit guiding principles are imprecise and likely to vary across experiments. Thus, we suggest that more effort should be devoted to defining stimulus features quantitatively rather than operationally. Quantitative definitions of features improve the ability to measure and control feature coverage and feature covariance. One substantial advantage of the voxel-wise modeling approach used here is that it provides a very clear and quantitative picture of what is known and what is not known. Stimulus properties can be quantified and modeled directly. Correlations between features within models and across models can also be quantified and assessed. This approach provides an unambiguous view of where the field is today, and it leads to clear recommendations for future studies.

### Conflict of interest statement

The authors declare that the research was conducted in the absence of any commercial or financial relationships that could be construed as a potential conflict of interest.
