# Supplementary material for: Fourier power, subjective distance, and object categories all provide plausible models of BOLD responses in scene-selective visual areas
Source: Front Comput Neurosci. 2015 Nov 5;9:135. doi: 10.3389/fncom.2015.00135 (PMC4633487; doi:10.3389/fncom.2015.00135)
Supplement: Supplementary file 1 [file Lescroart_2015_SOM.PDF]

Supplementary Online Material

Supplementary methods

To assess whether Fourier power was related to distance in two other studies (Kravitz, Peng, & Baker, 2011; Park, Konkle, & Oliva, 2014), we first obtained the full set of images used in each of the studies. Stimuli for Kravitz et al (2011) were obtained via correspondence with the senior author, and stimuli for Park et al were downloaded from Talia Konkle's web page (http://konklab.fas.harvard.edu/ImageSets/). All images in each experiment were processed with the same Fourier power model described in the Methods section of the main manuscript. For each experiment, before further analysis each Fourier power channel was first normalized to zero mean and unit variance (*z*-scored) across all the images in that experiment.

For the Kravitz, Peng, & Baker (2011) study, we used *t* tests to compare the mean Fourier power for each Fourier power channel in Open vs. Closed and Near vs. and Far conditions. However, the Near and Far conditions in Kravitz et al (2014) do not reflect the absolute or subjective distance to the main content of each scene. However, Kravitz et al did collect Elo ratings (Elo, 1978) for nearness for each of their scenes. These ratings indicate the relative perceived nearness of each scene on an arbitrary scale. Thus, we split the images into two groups based on the Elo ratings: Near (Elo) consisted of the half of the scenes with the lowest Elo ratings, and Far (Elo) consisted of the scenes with the largest Elo ratings. We used *t* tests to compare the mean of each Fourier channel for these relatively near and far scenes. Results of all condition comparisons are shown in Figure S09.

For the Park et al (2014) study, we created a binary label vector for each distance bin used in their study. These six distance label vectors are analogous to the five channels in our subjective distance model (they had six distance bins; we had five). We computed the correlation between each distance label and each Fourier power channel. Results are shown in Figure S10.

Supplementary figures

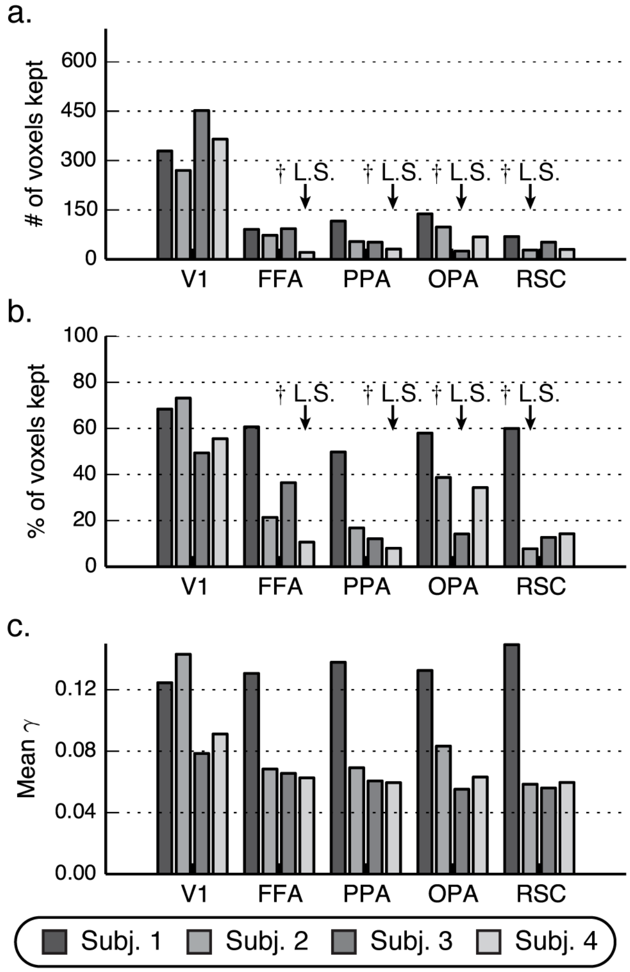

Figure S01: Voxel selection based on noise ceiling (*γ*). **a.** Absolute number of voxels retained per subject and per ROI (for $p(\gamma) < 0.01$). **b.** Percentage of voxels retained per subject and per ROI, using the same *γ* threshold as in panel a. **c.** Average *γ* across the voxels selected per subject and per ROI. Signal to noise varied substantially across subjects, and consequently some regions of interest in some subjects contained relatively few voxels. The †L.S. labels indicate the four regions with the lowest signal in the experiment (RSC in subject 2, OPA in subject 3, and FFA and PPA in subject 4). For each of these regions, the voxels selected by the γ threshold either numbered fewer than 30 voxels total, or constituted fewer than 10% of the voxels selected by the relevant localizer. Model fits in the specific subjects and areas that have low signal are less likely than the same areas in other subjects to reflect the true response properties of those areas.

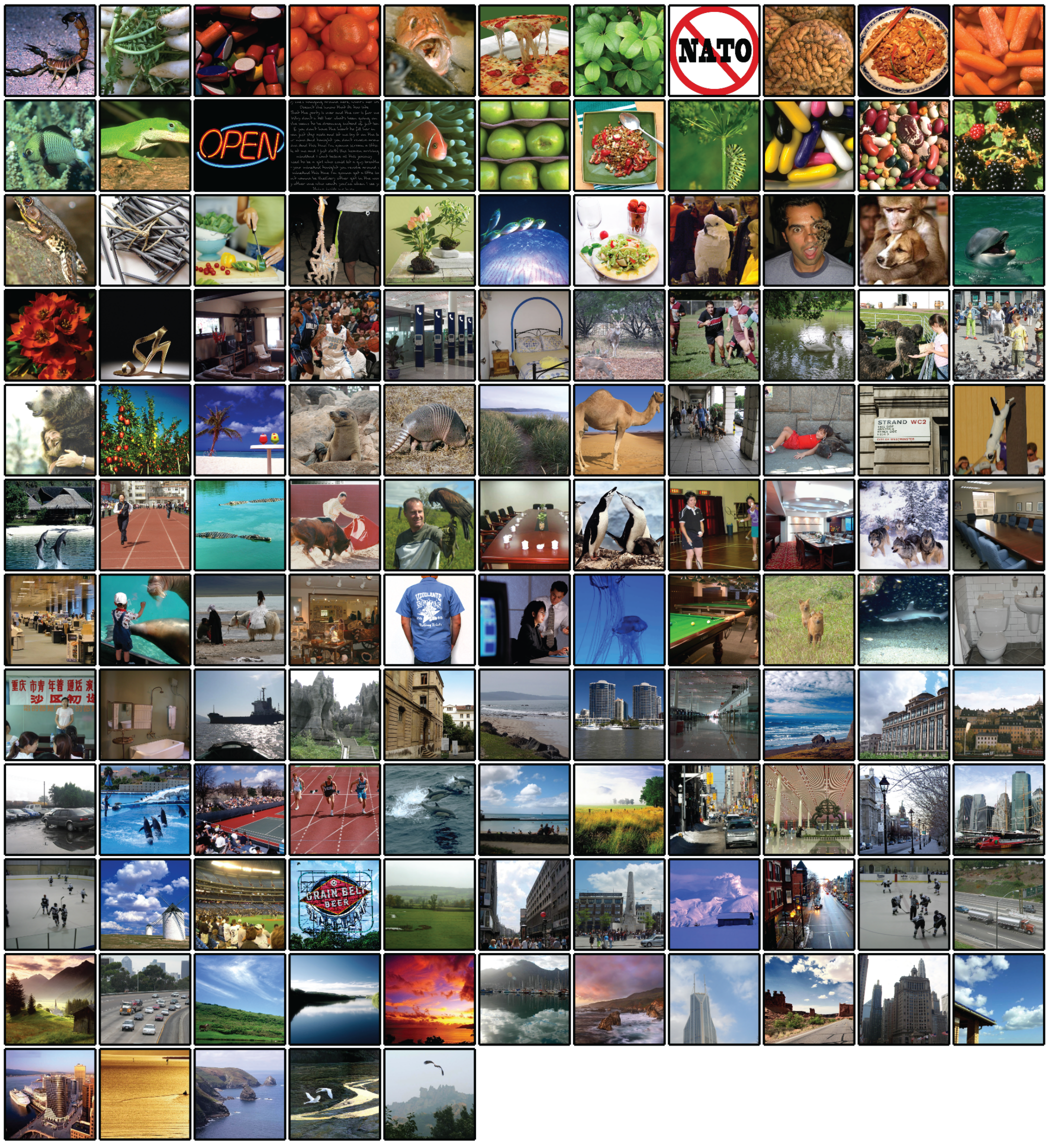

Figure S02: Images used in the validation set. Images are sorted from top left to bottom right by subjects' median distance rating. Images rated *Closeup (<1')* are located at the top and images rated *V. far (>100')* are at the bottom.

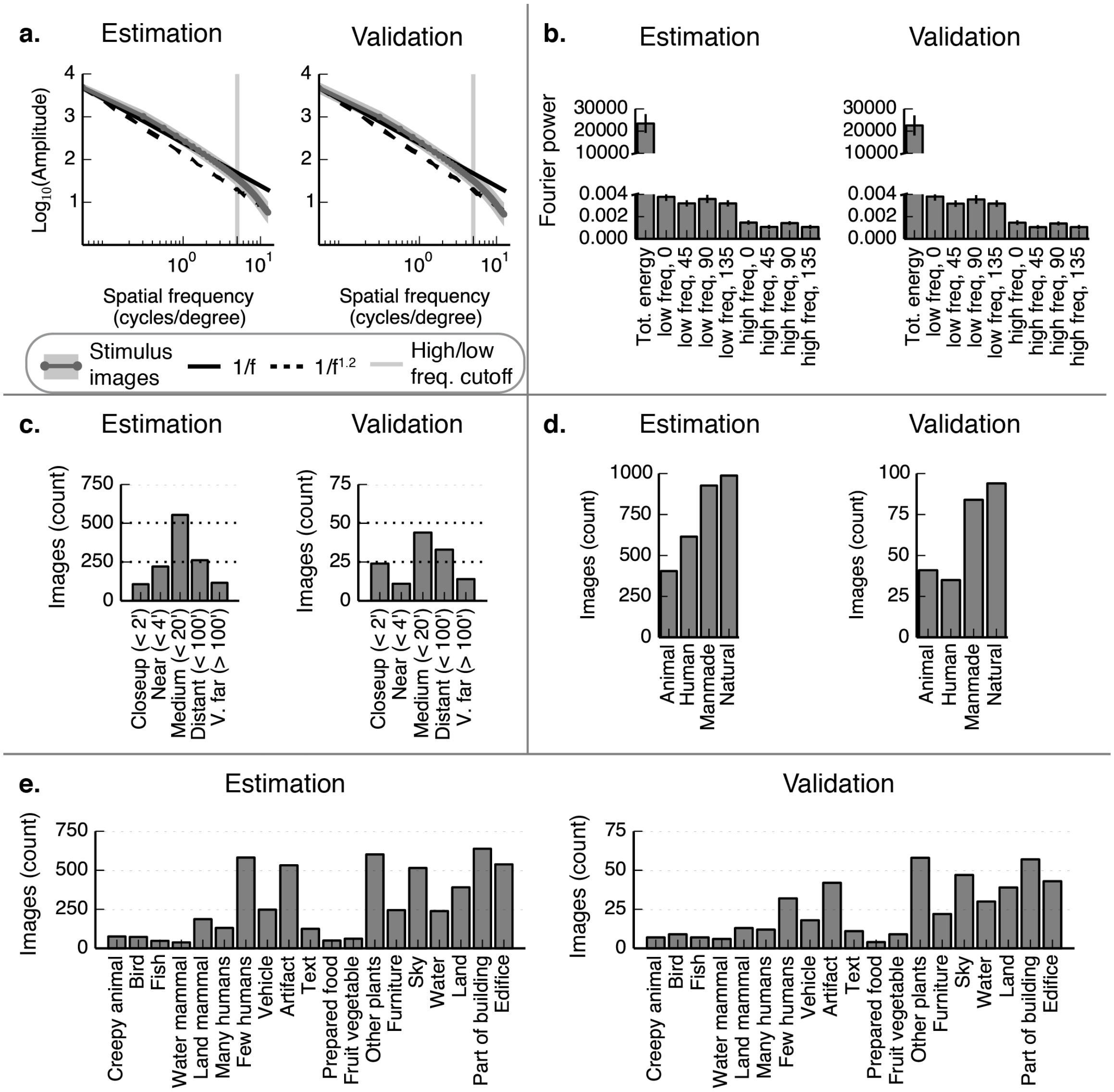

Figure S03: Statistical properties of the stimuli used in this study. **a.** Fourier power by spatial frequency, averaged over orientation, for estimation and validation stimulus sets. The faint gray fill shows the standard deviation over images. The vertical gray line shows the frequency used as a cutoff between high and low frequency channels in the Fourier power feature space. The highest spatial frequencies deviate slightly below the expected $1/f$ relationship between Fourier power and spatial frequency in natural images. This small deviation likely reflects blurring due to image compression and rescaling. These images were gathered from online sources, so it is impossible to determine how many times they may have been compressed/rescaled. **b.** Average values for the feature channels in the Fourier power model before z-scoring, for estimation and validation stimulus sets. **c.** Number of images in each of the subjective distance bins, for estimation and validation stimulus sets. **d.** Number of images labeled with each of four superordinate category labels. Images were selected for inclusion in the stimulus set based on these superordinate labels. An approximately equal number of images with the labels *animal* and *human* were selected, as well as an approximately equal number of images with the labels *manmade* and *natural*. **e.** Number of images with each object category label, for estimation and validation stimulus sets. For all stimulus properties measured, the distributions of feature values in the estimation stimulus set approximately match the distributions of features in the validation stimulus set.

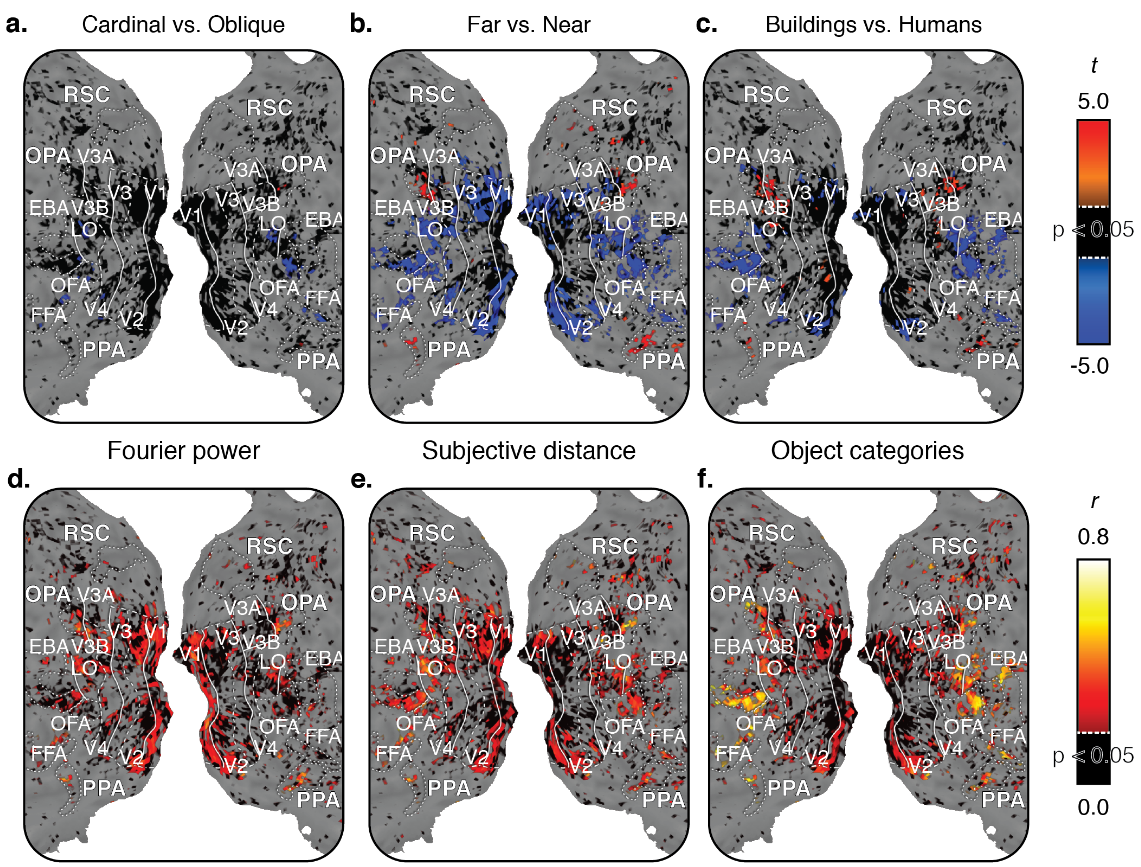

Figure S04: Maps of voxel-wise *t* contrasts and normalized prediction accuracy for subject 2. Conventions as in Figure 6 in the main text. For all maps, dashed lines indicate the horizontal meridian in the visual field, solid lines indicate the vertical meridian, and dotted lines indicate the boundaries of regions of interest defined by functional contrasts. *Note that this subject has low signal quality in RSC (Figure S01), which affects all plots in this figure.* **a.** *t* contrast computed for β weights within the Fourier power model (cardinal vs. oblique). *t* values are scaled from -5 to 5, black voxels indicate *t* values below the chance threshold ($t < 3.36$, FDR-corrected p>0.05) despite good signal ($\gamma > 0.04$, $p(\gamma) < 0.01$). Gray voxels indicate poor signal ($\gamma < 0.04$, $p(\gamma) > 0.01$) and thus no basis for comparing models. The absence of orange/red voxels (significantly positive *t* values) for this contrast indicates that this subject did not show the expected pattern of results. (Based on previous results the pre-determined *t* contrast tested for a difference between high-frequency vertical and horizontal orientations and all other orientations.) Examination of the β weights for this subject in Figure 5a, d, and g in the main text suggest that this occurred because both high and low-frequency vertical and horizontal orientations had similar β weights. **b.** *t* contrast for β weights within the subjective distance model (far vs. near). **c.** *t* contrast computed for β weights within the object category model (buildings vs. people). Voxels with significant *t* contrasts for the subjective distance and object category models are located in approximately the same regions of cortex. **d.** Prediction accuracy for the Fourier power model. Prediction accuracy has been normalized by the noise ceiling. Black voxels indicate correlations that are below the chance threshold ($r < 0.21$, FDR-corrected $p > 0.05$) despite good signal ($\gamma > 0.04$, $p(\gamma) < 0.01$). Gray voxels indicate poor signal ($\gamma < 0.04$, $p(\gamma) > 0.01$), and thus no potential to test predictions. **e.** Prediction accuracy for the subjective distance model. **f.** Prediction accuracy for the semantic category model. All three models make accurate predictions in similar locations across the cortex, though the object category model makes more accurate predictions in FFA, OFA, and EBA. This suggests that the three different models may each describe the same response variance in scene-selective areas in a different way. These results are broadly consistent across subjects (after taking into account differences in signal quality across subjects).

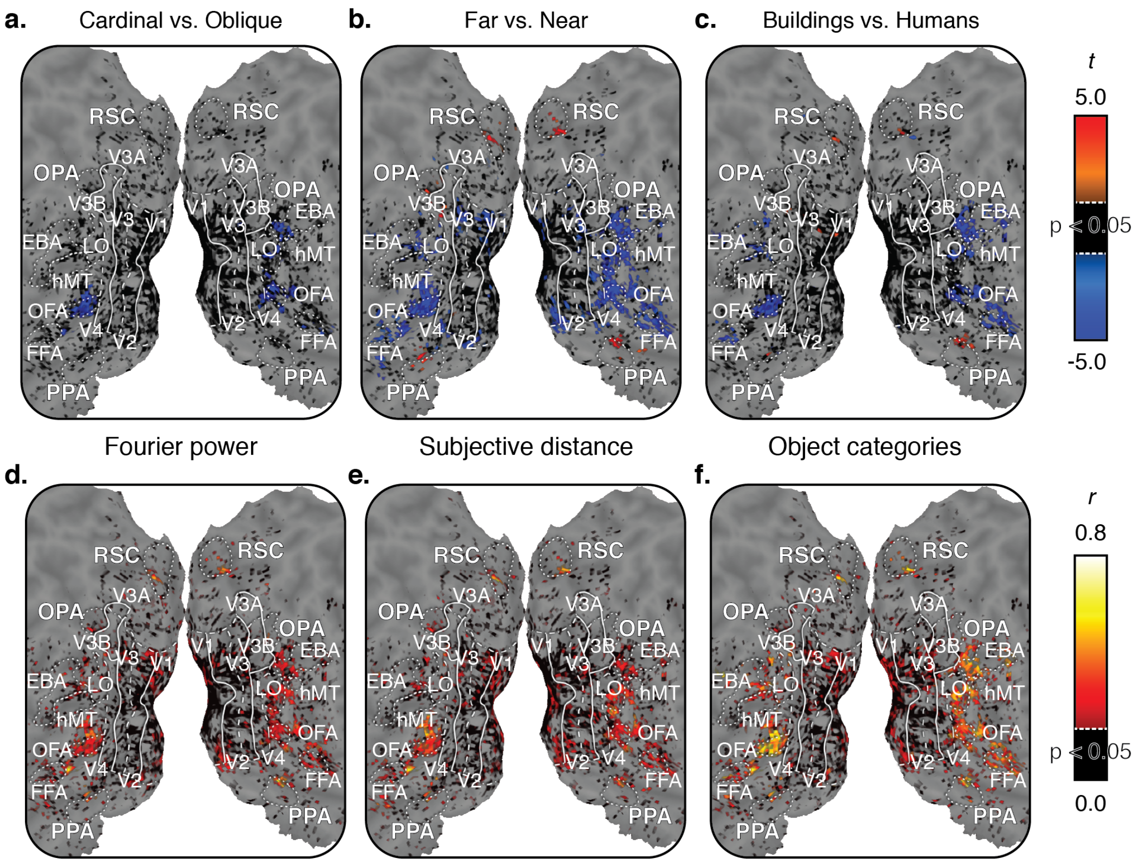

Figure S05: Maps of voxel-wise t contrasts and normalized prediction accuracy for subject 3. Conventions as in Figure 6 in the main text and Figure S04 above. *Note that this subject has low signal quality in OPA (Figure S01), which affects all plots in this figure.* **a.** *t* contrast computed for β weights within the Fourier power model (cardinal vs. oblique). As in subject 2, there are no orange/red voxels (significantly positive *t* values), so this subject did not show the expected pattern of tuning within the Fourier power model in any of the scene-selective areas. This could be because overall signal is poor in these areas (compared to subject 1). Despite the irregular patterns of tuning across subjects, the Fourier power model still makes accurate predictions in the voxels with adequate signal (see panel d. and Supplementary Figure 7). **b.** *t* contrast for β weights within the subjective distance model (far vs. near). **c.** *t* contrast computed for β weights within the object category model (buildings vs. humans). Voxels with significant *t* contrasts for the subjective distance and object category models are located in the approximately the same regions of cortex. **d.** Prediction accuracy for the Fourier power model. **e.** Prediction accuracy for the subjective distance model. **f.** Prediction accuracy for the semantic category model. All three models make accurate predictions in similar locations across the cortex, though the object category model makes more accurate predictions in FFA, OFA, and EBA. These results are broadly consistent across subjects (after taking into account differences in signal quality across subjects).

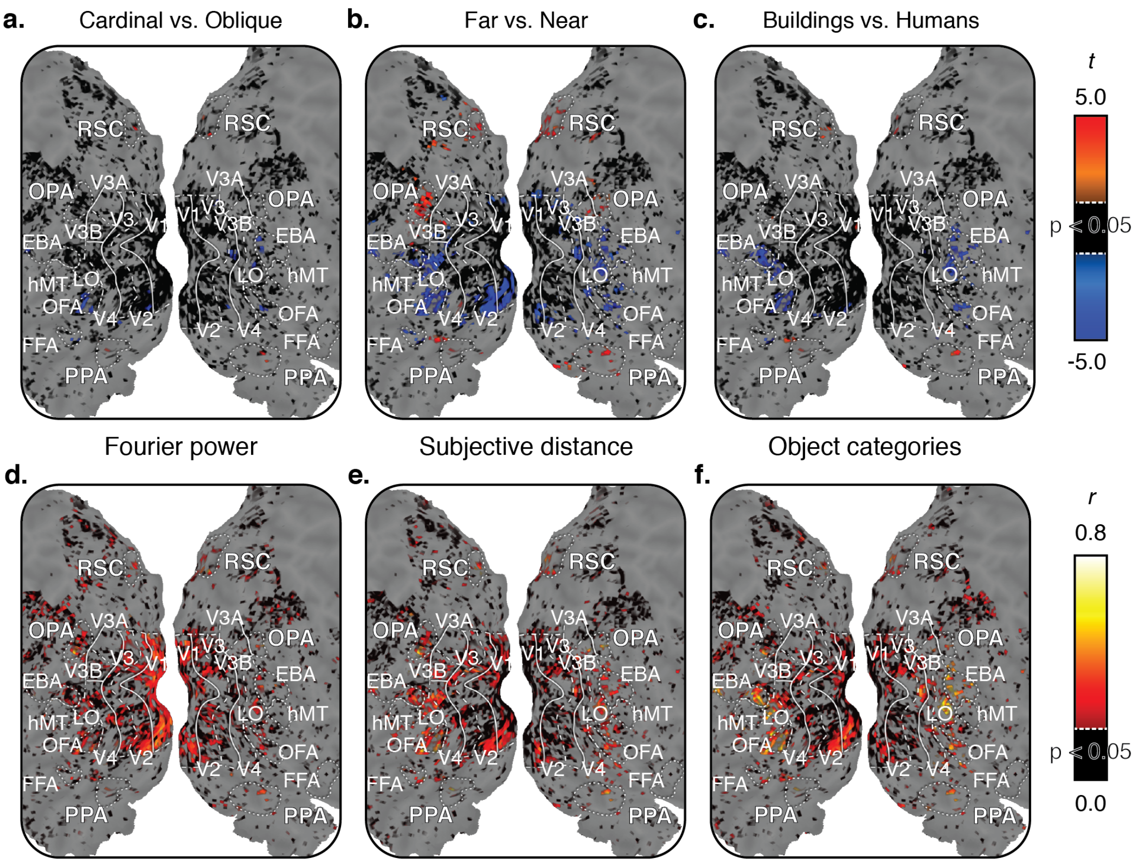

Figure S06: Maps of voxel-wise t contrasts and normalized prediction accuracy for subject 4. Conventions as in Figure 6 in the main text and Figure S04 above. *Note that this subject has low signal quality in PPA and FFA (Figure S01), which affects all plots in this figure.* **a.** *t* contrast computed for β weights within the Fourier power model (cardinal vs. oblique). As in subjects 2 and 3, there are no (or very few) orange/red voxels (significantly positive *t* values). This indicates that this subject did not show the expected pattern of tuning within the Fourier power model in any of the scene-selective areas. This could be because overall signal is poor in these areas (compared to subject 1). Despite the irregular patterns of tuning across subjects, the Fourier power model still makes accurate predictions in the voxels with adequate signal (see panel d. and Supplementary Figure 7). **b.** *t* contrast for β weights within the subjective distance model (far vs. near). **c.** *t* contrast computed for β weights within the object category model (buildings vs. humans). For this subject, OPA does not contain any voxels with significant contrasts for buildings vs. humans, despite adequate signal. As noted in the main text, the building vs. humans contrast was pre-determined based on prior experiments, and does not reflect the full tuning profile revealed by the semantic model. This and all other *t* contrasts are only intended to provide a familiar analysis as a reference point to compare to other studies. The weights plotted in for subject 4 in Figure 5i in the main text show that across the full set of object category labels, tuning in OPA in subject 4 was consistent with other subjects. Outside of OPA, voxels with significant *t* contrasts for the subjective distance and object category models are located in approximately the same regions of the cortex. **d.** Prediction accuracy for the Fourier power model. **e.** Prediction accuracy for the subjective distance model. **f.** Prediction accuracy for the semantic category model. All three models make accurate predictions in similar locations across the cortex, though the object category model makes more accurate predictions in OFA and EBA. These results are broadly consistent across subjects (after taking into account differences in signal quality across subjects).

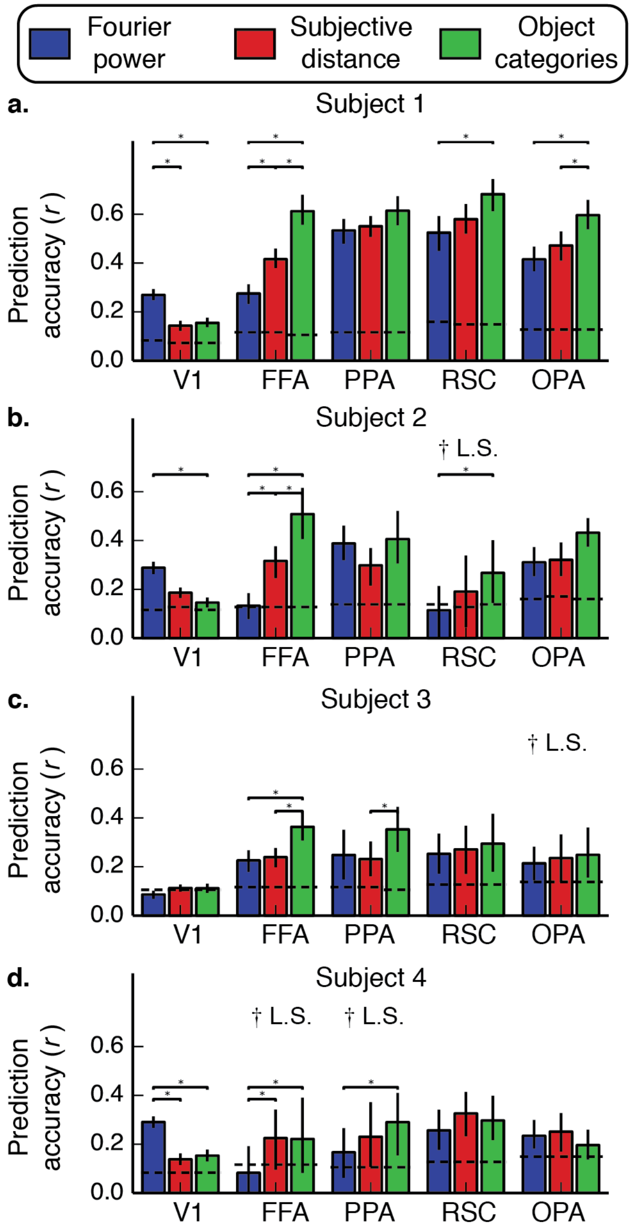

Figure S07: Prediction accuracy for each model, each ROI and each individual subject, analogous to Figure 7 in the main text. Predictions are normalized by the noise ceiling. Only voxels with reliable stimulus-evoked responses are included. Error bars are 99% confidence intervals. Asterisks indicate significant differences between models (bootstrapped $p < 0.05$). The dotted lines across the bottom indicate the chance threshold (bootstrapped $p = 0.05$) for the mean correlation for each ROI. (Thresholds differ slightly across ROIs because of the differing number of voxels in each ROI.) †L.S. labels denote ROIs with low signal quality (see Supplementary Figure 1). The data in these ROIs are less likely than the same regions in other subjects to reflect true response properties or model accuracy. **a.** Prediction accuracy for subject 1. **b.** Prediction accuracy for subject 2. Prediction accuracy for the Fourier power model does not reliably exceed chance in RSC. RSC had low signal quality in this subject (Figure S01). Voxel selection based on the noise ceiling (γ) only chose 8% of the voxels (28/359 voxels) in RSC. **c.** Prediction accuracy for subject 3. **d.** Prediction accuracy for subject 4. Both FFA and PPA had poor signal quality in this subject (Figures S01). Voxel selection based on the noise ceiling (γ) only chose 11% of the voxels (21/197) in FFA and 8% of the voxels (31/387 voxels) in PPA. The low signal quality in FFA is likely to be the reason that the object category model did not out-perform the other models in FFA. Thus, prediction accuracy was above chance for all three models for all subjects in all three scene-selective areas, except in the regions with low signal. Among all the scene-selective areas, none of the three models makes reliably more accurate predictions than any other model across subjects.

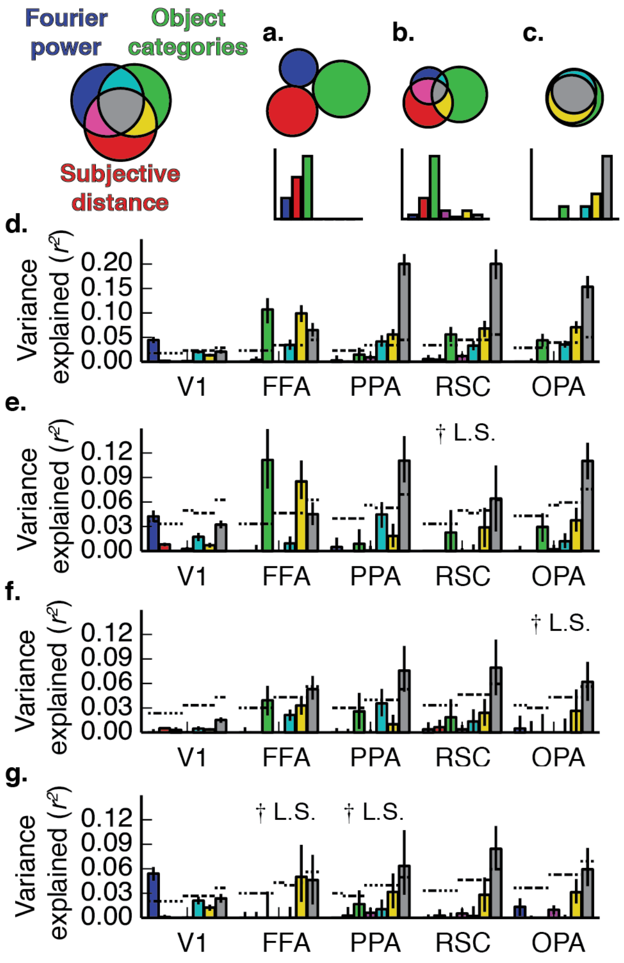

Figure S08: Three-way variance partitioning analysis, for each model, subject, and ROI, analogous to Figure 8 in the main text. **a.** Venn diagram representing hypothetical relationships between the variance explained by the three models, such that each explains a unique component of the variance. **b.** A different hypothetical relationship in which the semantic category model explains a large fraction of independent variance, but the three models all share small amounts of variance. **c.** A third possible relationship in which all three models explain shared variance, and the Fourier power and subjective distance models account for most of the variance explained by the object category model. **d.** Three-way variance partitioning results for subject 1. Dotted lines at the bottom of the graph indicate chance thresholds (bootstrapped p = 0.05) for the amount of variance explained. Chance thresholds differ depending on the number of voxels per ROI and the number of subtractions between fit models necessary to compute each component of the variance. **e.** Three-way variance partitioning results for subject 2. †L.S. labels denote regions with low signal. **f.** Three-way variance partitioning results for subject 3. **g.** Three-way variance partitioning results for subject 4. Note that this subject had particularly poor signal in FFA. Across subjects, after accounting for differences in signal to noise across subjects, the pattern of results is most consistent with the Venn diagram in c.

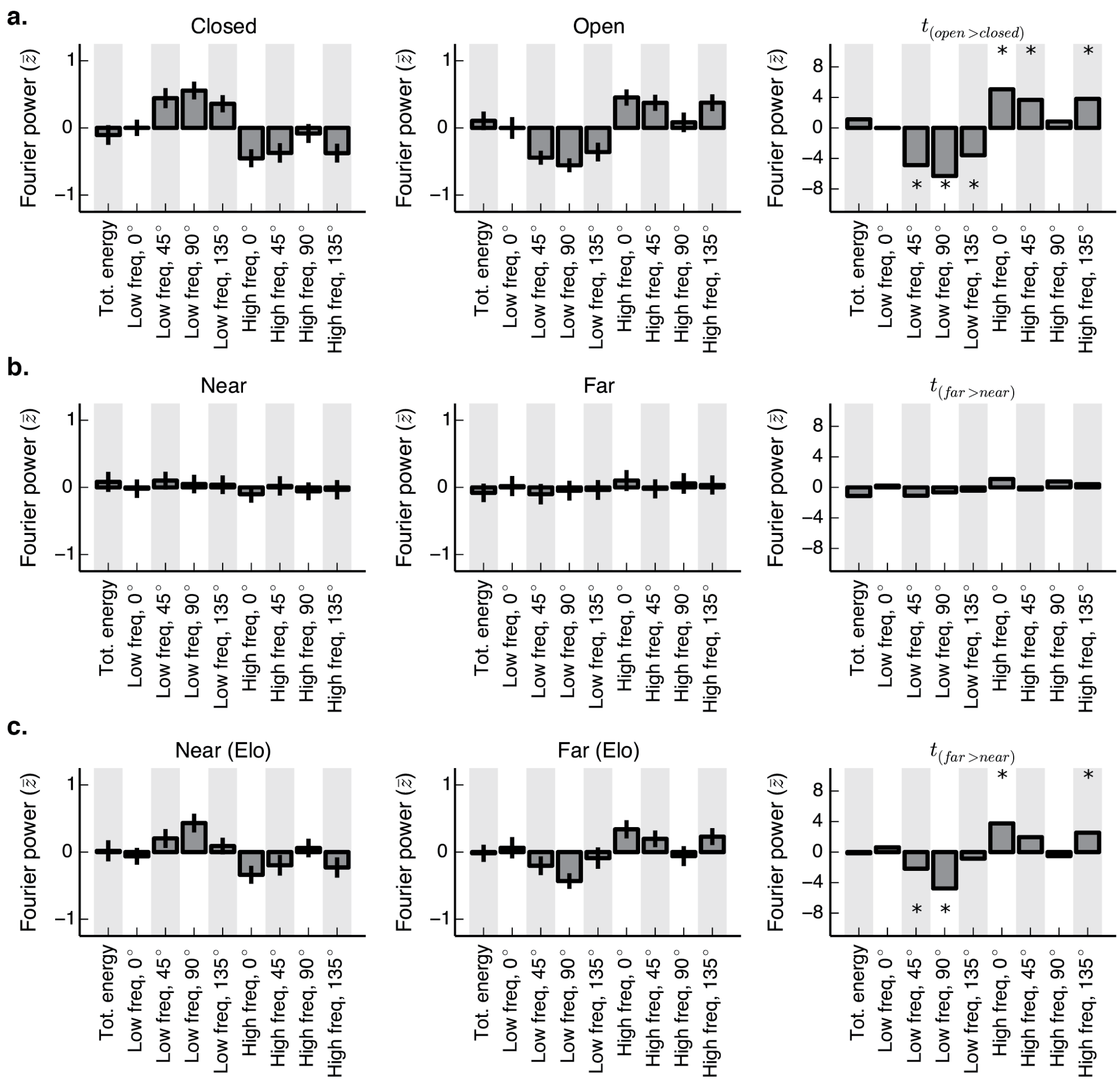

Figure S09: Relationship between Fourier power and the Expanse and Distance categories from (Kravitz et al., 2011). **a.** Mean z-scored Fourier power for Open and Closed experimental categories and a plot of the t values computed for the difference between open and closed scenes. Open and closed scenes differ reliably in low frequency vertical and oblique Fourier power, as well as in high frequency horizontal and oblique Fourier power. **b.** Mean z-scored Fourier power for Near and Far experimental categories and a plot of the t values computed for the difference between near and far scenes. Scenes with the condition labels Near and Far do not reliably differ in any Fourier power channel. **c.** The Near and Far conditions labels in Kravitz et al (2014) do not reflect absolute distance to the main content of each scene. Thus, we used the Elo ratings obtained in Kravitz, Peng, & Baker to divide the scenes into those rated nearest and farthest by human subjects. The three plots here show the mean z-scored Fourier power for the half of the scenes judged to be nearest and the half of the scenes judged to be farthest away, as well as t values computed for the difference between scenes rated as far and near. Scenes rated as near and far differ reliably in low frequency vertical Fourier power and in high frequency horizontal Fourier power.

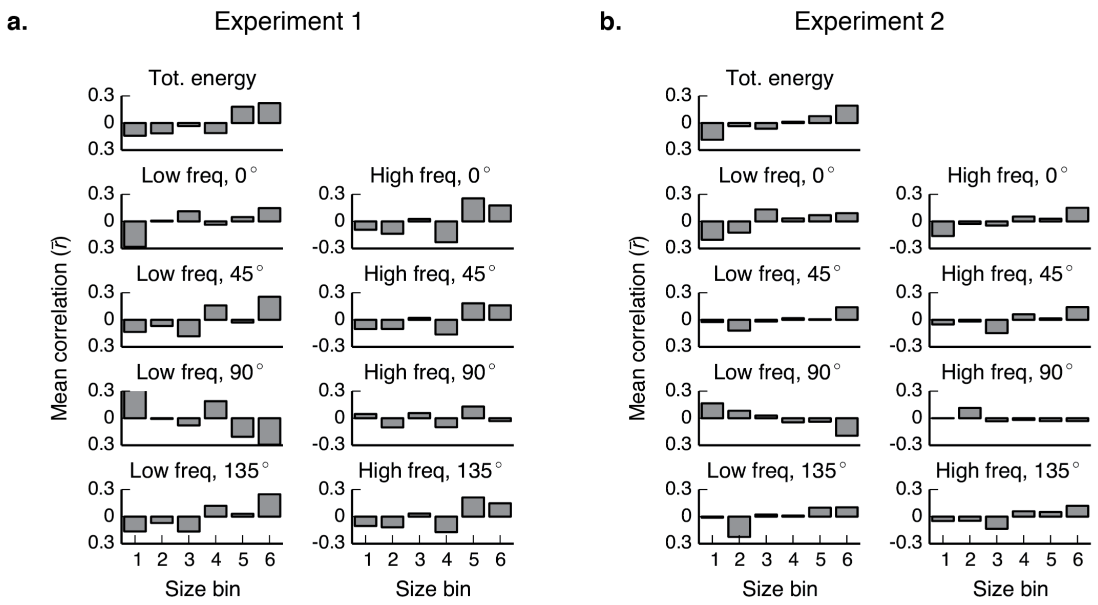

Figure S10: Correlations between Fourier power and distance in Park, Konkle, & Oliva (2014). These plots are analogous to those shown in Figure 10b and c in the main text. **a.** Correlations between each Fourier power channel and each distance bin for the stimuli in Park et al's Experiment 1. **b.** Correlations between each Fourier power channel and each distance bin for the stimuli in Park et al's Experiment 2. For both experiments, far distances are correlated with high-frequency horizontal Fourier power and near distances are correlated with vertical low frequency Fourier power, just as in our data.

Supplementary Bibliography

Elo, A. E. (1978). *The rating of chess players, past and present*. New York: Arco Publishing.

Kravitz, D. J., Peng, C. S., & Baker, C. I. (2011). Real-world scene representations in high-level visual cortex: it's the spaces more than the places. *The Journal of Neuroscience : The Official Journal of the Society for Neuroscience*, *31*(20), 7322–33. doi:10.1523/JNEUROSCI.4588-10.2011

Park, S., Konkle, T., & Oliva, A. (2014). Parametric Coding of the Size and Clutter of Natural Scenes in the Human Brain. *Cerebral Cortex (New York, N.Y. : 1991)*. doi:10.1093/cercor/bht418

Appendix 1: Subject instructions for subjective distance ratings

In this rating session, you will view a series of images of scenes. Each image / scene will appear only briefly. For each scene, your task is to determine how far away the main content in the scene is. It is up to you to determine what the "main content" is. Try to get a sense for what is going on in the scene, and base your judgment on whatever appears to be most salient / important; do not simply choose the minimum or maximum distance visible. You will rate the distance as falling into one of five bins (indicated by pressing 1-5 on the keyboard):

1 = extreme closeup (~1-2 ft)
2 = arm's length (~3-4 ft)
3 = nearby / same room (<20 ft)
4 = semi-distant (< 100 ft)
5 = far away (> 100 ft)

For each trial, you will initiate the trial by pressing any keyboard key, and then press 1-5 once you have made your judgment. Press 6 to repeat a trial, but only use this if you feel you have made a mistake (pressed 1 when you meant 2). Each rating should be based on your initial impression, not on repeated views.
